# A study on the association between gut microbiota, inflammation, and type 2 diabetes

**DOI:** 10.1007/s00253-024-13041-5

**Published:** 2024-02-15

**Authors:** Nannan Liu, Xuehua Yan, Bohan Lv, Yanxiang Wu, Xuehong Hu, Chunyan Zheng, Siyu Tao, Ruxue Deng, Jinfang Dou, Binfang Zeng, Guangjian Jiang

**Affiliations:** 1 Laboratory of Diabetes Research Center, College of Traditional Chinese Medicine, Beijing University of Chinese Medicine, No. 11, Bei San Huan Dong Lu, Beijing, 100029 Chaoyang District China; 2 College of Traditional Chinese Medicine, Xinjiang Medical University, No.393 Xin Medical Road, Urumqi, 830011 Xinjiang China

**Keywords:** Retinoic acid-inducible gene I-like receptors, Gut microbiota, Inflammation, Type 2 Diabetes mellitus, Tryptophan metabolism, Multi-omics co-analysis

## Abstract

**Abstract:**

Type 2 diabetes mellitus (T2DM) was reported to be associated with impaired immune response and alterations in microbial composition and function. However, the underlying mechanism remains elusive. To investigate the association among retinoic acid-inducible gene-I-like receptors (RLRs) signaling pathway, intestinal bacterial microbiome, microbial tryptophan metabolites, inflammation, and a longer course of T2DM, 14 patients with T2DM and 7 healthy controls were enrolled. 16S rRNA amplicon sequencing and untargeted metabolomics were utilized to analyze the stool samples. RNA sequencing (RNA-seq) was carried out on the peripheral blood samples. Additionally, C57BL/6J specific pathogen-free (SPF) mice were used. It was found that the longer course of T2DM could lead to a decrease in the abundance of probiotics in the intestinal microbiome. In addition, the production of microbial tryptophan derivative skatole declined as a consequence of the reduced abundance of related intestinal microbes. Furthermore, low abundances of probiotics, such as *Bacteroides* and *Faecalibacterium*, could trigger the inflammatory response by activating the RLRs signaling pathway. The increased level of the member of TNF receptor-associated factors (TRAF) family, nuclear factor kappa-B (*NF-κB*) activator (*TANK*), in the animal colon activated nuclear factor kappa B subunit 2 (*NFκB2*), resulting in inflammatory damage. In summary, it was revealed that the low abundances of probiotics could activate the RLR signaling pathway, which could in turn activate its downstream signaling pathway, NF-κB, highlighting a relationship among gut microbes, inflammation, and a longer course of T2DM.

**Key points:**

- *Hyperglycemia may suppress tryptophanase activity.*
- *The low abundance of Bacteroides combined with the decrease of Dopa decarboxylase (DDC) activity may lead to the decrease of the production of tryptophan microbial derivative skatole, and the low abundance of Bacteroides or reduced skatole may further lead to the increase of blood glucose by downregulating the expression of glucagon-like peptide-1 (GLP1).*
- *A low abundance of* anti-inflammatory bacteria *may induce an inflammatory response by triggering the RLR signaling pathway and then activating its downstream NF-κB signaling pathway in prolonged T2DM.*

**Supplementary Information:**

The online version contains supplementary material available at 10.1007/s00253-024-13041-5.

## Introduction

The incidence and prevalence of type 2 diabetes mellitus (T2DM) are on the rise globally (Khosla et al. 2021), constituting approximately 90% of all diabetic cases (Kerner and Brückel 2014). Moreover, T2DM is accompanied by chronic, low-grade inflammation, impaired immune response, and changes in the composition and function of the gut microbiome (Gutiérrez-Salmerón et al. 2021). Conversely, inflammation can disrupt gut structure, leading to insulin resistance (IR) and reduced insulin secretion (Dyer et al. 2020; Rohm et al. 2022). Additionally, intestinal microorganisms can induce metabolic dysfunction and compromise the barrier function of the intestinal epithelium through their own activities or metabolites. This may affect the mucosal immune response of the intestines (Sittipo et al. 2018). Researchers have demonstrated that retinoic acid-inducible gene-I-like receptors (RLRs) function as pattern recognition receptors (PRRs), which are responsible for the body’s first line of defense against pathogenic microorganisms (Wicherska-Pawłowska et al. 2021). Previous studies have indicated that RLRs can induce innate immunity by identifying and combining microbe-associated molecular patterns (MAMPs) (Medzhitov 2007). Furthermore, they can initiate the RLRs signaling pathway (Kelly et al. 2004), inducing microbicidal activities that can change the composition of the gut microbiome (Privitera et al. 2021). Studies have found that commensal anaerobic bacteria in the gut can attenuate the inflammatory response by regulating NF-κB subunit p65 (*RELA*) of the RLRs signaling pathway. Moreover, excessive activation of RLR signaling is implicated as a pivotal factor in either triggering or facilitating the onset of autoinflammation (Zhao et al. 2017). Additionally, the microbiome serves as an extensive chemical factory, producing numerous compounds essential for its own sustenance and that of its hosts. Given that these compounds can reach the liver through the portal circulation system (Saeedi et al. 2020) and subsequently circulate throughout the body via hepatic veins, capillaries, etc., they exert a remarkable influence on the entire holobiont (Fan and Pedersen 2021). Research indicated that the intestinal microbiome is linked to diverse diseases, including metabolic syndrome, diabetes, inflammation, and cardiovascular diseases (Nicholson et al. 2012).

Tryptophan stands as an indispensable amino acid that is crucial for the human body, with its sole dietary source being derived from food intake. Approximately 4–6% of tryptophan undergoes direct metabolism by gut microbiota (Yokoyama and Carlson 1979). Furthermore, the metabolism of tryptophan plays a pivotal role in shaping the diversity of the intestinal microbiome (Gao et al. 2018). Studies have highlighted the anti-inflammatory properties of tryptophan metabolites, such as indole and skatole, in mammals (Marsland 2016). Notably, these microbial derivatives of tryptophan activate signaling pathways regulating the differentiation and function of various immune cells, including T cells (Liu et al. 2018), B cells (Piper et al. 2019), innate immune cells (ILCs) (Zelante et al. 2014), and CD4+CD8αα+ lymphocytes (Cervantes-Barragan et al. 2017). This activation contributes to sustaining the dynamic balance and functionality of immune cells (Peterson and Artis 2014). Research has demonstrated that rats subjected to a low-tryptophan diet exhibited an increased susceptibility to inflammation (Hashimoto et al. 2012). Yin and Yang deficiency syndrome, a characteristic of traditional Chinese medicine (TCM) syndrome associated with IR, is notably prevalent in patients with T2DM (Xie et al. 2011). A clinical trial revealed that, in comparison to other T2DM syndromes, the proportion of patients with Yin and Yang deficiency syndrome was the lowest, and those patients exhibited an older average age. Distinctive clinical symptoms, such as thirst, frailty, nocturnal polyuria, soreness, and weakness in the knees and waist, differentiate Yin and Yang deficiency syndrome from other syndromes of T2DM (Zhang et al. 2021). This suggests that the Yin and Yang deficiency syndrome may represent a prolonged course of T2DM.

Recent research demonstrated that the microbial tryptophan derivative, indole, has the potential to regulate glucagon-like peptide-1 (GLP1) expression. This regulatory action contributes to reducing fasting plasma glucose (FPG) levels by stimulating pancreatic β cells to secrete insulin through the activation of enteroendocrine L cells. Moreover, microbial tryptophan metabolites play a crucial role in shaping the composition of the intestinal microbiome and influencing the immune response in the intestinal mucosa. This leads to notable anti-inflammatory effects and the modulation of interactions between the intestinal microbiome and inflammatory responses (Nicholson et al. 2012). Specifically, *Bacteroides* and *Faecalibacterium* have been reported to attenuate the inflammatory response by impacting the RLRs signaling pathway through RELA (Cervantes-Barragan et al. 2017; Kelly et al. 2004). Furthermore, a study indicated that both native immune cells and adaptive immune cells establish intricate connections among T2DM, gut microbiota, and immune dysfunction (Rohm et al. 2022). It is noteworthy that PRRs, located in the cell membrane or cytoplasm, are essential prerequisites for immune cells to initiate an immune response (Ori and Kawai 2023). Remarkably, despite the intricate interplay among gut microbiota, microbial tryptophan metabolites, prolonged courses of T2DM, and the innate immune response with RLRs as a focal point, no research has comprehensively examined this relationship. Therefore, the present study aimed to reveal this complex relationship, employing bacterial gut microbiome, untargeted metabolomics, and RNA sequencing (RNA-seq) through a multi-perspective and integrated analysis. This research may provide a foundation for potential therapeutic interventions for extended courses of T2DM.

## Materials and methods

### Human subjects

This study and experimental protocol have been approved by the Ethics Committee of Beijing University of Chinese Medicine (BUCM) under approval number #2017BZHYLL0105. Prior to enrolment, each participant signed a written informed consent form.

The research involved 14 patients with T2DM and seven healthy controls, all recruited from Hepingli Hospital, Beijing, China. After that, the T2DM patients were then categorized into two groups: the YYLXD group (IDs: YYLXD001, YYLXD002, YYLXD003, YYLXD004, YYLXD005, YYLXD006, and YYLXD008), and the NYYLXD group (IDs: QYLXD001, QYLXD002, QYLXD003, SRKPD001, SRKPD002, YXRSD001, and YXRSD002). The healthy participants formed the normal (N) group (IDs: ZC001, ZC002, ZC003, ZC004, ZC005, ZC006, and ZC007). Each T2DM patient fulfilled diagnostic criteria with an FPG level of ≥ 7.0 mmol/L and a hemoglobin A1C (HbA1C) level of ≥ 6.5%. Additionally, following the guidelines for clinical research on new Chinese medicines for diabetes treatment set forth by the Ministry of Health of China in 2002 (Zhang et al. 2021), T2DM patients underwent simultaneous evaluation by three clinical associate chief and director physicians. This comprehensive assessment considered clinical symptoms, tongue appearance, and pulse patterns and ensured the absence of complications. Subsequently, these patients were categorized into distinct TCM syndromes based on referenced literature (Xu et al. 2013; Yang et al. 2010). Conversely, healthy subjects displayed no history of metabolic diseases, maintained standard vital signs, and exhibited normal screening laboratory results (e.g., FPG, HbA1c, total cholesterol (TC), triglycerides (TG), low-density lipoprotein cholesterol (LDL-C), and high-density lipoprotein cholesterol (HDL-C)). No antibiotics were utilized in the preceding three days, and no chronic conditions, such as heart failure, pulmonary disease, renal failure, or gastrointestinal disorders, occurred.

Venous blood and human stool samples were collected from both healthy volunteers and T2DM patients. The peripheral venous blood underwent laboratory analysis, while the human stool samples were promptly frozen and transported on dry ice to the Novegene Laboratory (Beijing, China). Subsequently, they were stored at −80 °C for metabolite and DNA extraction.

### Experimental animals

All animal procedures and experimental protocols received approval from the Ethics Committee of the BUCM. For the experiments, age-matched C57BL/6J SPF mice were utilized, which were attained from Beijing Vital River Laboratory Animal Technology (Beijing, China). All mice were aged between 4–6 weeks upon the commencement of the study. They were housed under specific pathogen-free conditions at BUCM, maintained at a temperature of 20–24 °C, with a 12-h light-dark cycle, 35% ± 5% humidity, and granted unrestricted access to tap water. Their diet consisted of a standard rodent feed (Cat#1022, Beijing Huafukang Biotechnology, Beijing, China).

The experiments involved a total of 16 mice, with eight mice subjected to an 8-week high-fat diet (HFD) to induce IR, forming the model (M) group. The remaining eight mice, consuming a regular rodent diet (Cat#1022, Beijing Huafukang Biotechnology, Beijing, China), comprised the normal group (N). After a 12-h fasting period, mice from the model group received an intraperitoneal injection of freshly prepared streptozotocin (STZ) (dissolved in 0.1 M citrate buffer, pH 4.5) for 24 h to induce mild insulin secretion deficiency and subsequent hyperglycemia. The control group received equivalent volumes of saline injections. After 7 days, tail vein blood sampling was performed to examine the FPG level. Mice with an FPG level exceeding 11.1 mmol/L after 6 h were identified as diabetic. Throughout the experimental period, the FPG level was routinely monitored at bi-weekly intervals.

### 16S rRNA gene amplicon sequencing

Genomic DNA was extracted from human stool samples using the cetyltrimethylammonium bromide (CTAB) method (Bulgarelli et al. 2015), and the concentration and purity of the extracted DNA were assessed on 1% agarose gels. Depending on its concentration, the DNA was diluted to 1 ng/μL with sterile water. Specific primers (e.g., 16S V4: 515F806R) with a barcode were employed to amplify distinct subregions of the 16S rRNA genes. All PCR reactions were performed with 15 μL of Phusion® High-Fidelity PCR Master Mix (New England Biolabs, Ipswich, MA, USA), 2 μM of forward and reverse primers (Hess et al. 2011; Youssef et al. 2009), and approximately 10 ng template DNA. The thermal cycling was commenced with an initial denaturation at 98 °C for 1 min, followed by 30 cycles of denaturation at 98 °C for 10 s, annealing at 50 °C for 30 s, and elongation at 72 °C for 30 s. Finally, the last step was at 72 °C for 5 min. The same volume of 1× sample loading buffer was mixed with PCR products and the mixture was loaded onto a 2% agarose gel for detection by electrophoresis. The agarose band containing the mixture of PCR products was purified using a Qiagen gel extraction kit (Qiagen, Hilden, Germany). Sequencing libraries were generated via the TruSeq® DNA PCR-free sample preparation kit (Illumina, San Diego, CA, USA) following the manufacturer’s instructions, and index codes were incorporated. The final library was sequenced on an Illumina NovaSeq platform, producing 250-bp paired-end reads.

### Untargeted metabolomics

Fecal samples (5–10 g) from subjects were collected in the morning. These samples were individually ground with liquid nitrogen, and the homogenate was resuspended with prechilled 80% methanol by vortexing. After incubation on ice for 5 min, the samples were centrifuged at 15,000 g for 20 min at 4 °C. A portion of the supernatant was diluted to achieve a final 53% methanol concentration using chromatography-mass spectrometry (LC-MS) grade water. The samples were subsequently transferred to a fresh Eppendorf tube and centrifuged at 15,000g for 20 min at 4 °C. The resulting supernatant was analyzed using the LC-MS/MS system. Ultra high-performance liquid chromatography (UHPLC)-MS/MS analysis was conducted using a Vanquish UHPLC system (Thermo Fisher Scientific, Waltham, MA, USA) coupled with an Orbitrap Q ExactiveTMHF mass spectrometer (ThermoFisher, Braunschweig, Germany). Raw data files generated by UHPLC-MS/MS were processed using Compound Discoverer 3.1 (CD3.1, Thermo Fisher Scientific) for peak alignment, peak picking, and quantitation of each metabolite. Metabolites were annotated using the KEGG (https://www.genome.jp/kegg/pathway.html), HMDB (https://hmdb.ca/metabolites), and LIPIDMaps (http://www.lipidmaps.org/) databases*.*

### RNA-seq of human peripheral blood

Notably, 7–8 ml of fasting venous blood was collected from each subject and dispensed into ethylenediaminetetraacetic acid (EDTA) anticoagulation tubes. The RNA degradation and contamination were monitored on 1% agarose gels. The purity of the RNA was determined using the NanoPhotometer® spectrophotometer (IMPÜLEN. Westlake Village, CA, USA). The integrity of the RNA was verified using the RNA nano 6000 assay kit of the Bioanalyzer 2100 system (Agilent Technologies, Santa Clara, CA, USA). For RNA sample preparation, 1 μg of RNA from each sample was utilized. Sequencing libraries were generated using NEBNext®UltraTM RNA library prep kit for Illumina® (NEB, Ipswich, MA, USA) on the basis of the manufacturer’s protocol, and specific index codes were added to link sequences to their respective samples. The clustering of the index-coded samples was performed on a cBot Cluster Generation system using TruSeq PE Cluster kit v3-cBot-HS (Illumina, San Diego, CA, USA) according to the manufacturer’s instructions. After clustering, the library preparations were sequenced on an Illumina NovaSeq platform, and 150-bp paired-end reads were generated.

### Histological assessment

After embedding, sectioning, and dewaxing, the colon, liver, and kidney tissues were stained with hematoxylin and eosin (H&E). The pathological characteristics of the liver, kidney, and colon tissues were then scrutinized under a microscope (magnification × 20, U-LH100-3, Olympus, Tokyo, Japan). Finally, we performed an inflammation score concerning this literature (Cheng et al. 2008; Rank et al. 1995).

### Western blot analysis of murine colon

Subsequently, 50 mg of colon tissue was homogenized in 500 μL of radioimmunoprecipitation assay (RIPA) lysate with protease inhibitor, and the final homogenate was collected. After centrifugation at 12,000 rpm for 5 min at 4 °C, the clear supernatant was collected for further analysis. Protein concentrations were determined using the bicinchoninic acid assay (BCA) kit (LABLEAD, Beijing, China). Thereafter, 20 μg of protein for each sample was mixed with 5× loading buffer, respectively, and boiled for 5 min. Separated proteins were transferred to polyvinylidene fluoride (PVDF) membranes, and they were subsequently blocked with 5% skimmed milk in Tween-20 (TBST) for 1.5 h at room temperature. After thrice washing with 1× Tris-buffered saline with TBST for 10 min each, the PVDF membranes were incubated with primary antibodies [rabbit anti-TANK (1:1000; ABclonal, Woburn, MA, USA) and rabbit anti-NFκB2 (p100/p52) (1:600; Proteintech. Rosemont, IL, USA)] overnight at 4 °C. Following additional thrice washing with 1× TBST, the PVDF membranes were incubated with the corresponding secondary antibodies at room temperature for 1 h. After extensive washing with 1× TBST, proteins were visualized by enhanced chemiluminescence (ECL) detection. Densitometric analysis was carried out using the Image J software.

### Immunohistochemistry (IHC)

The colon, liver, and kidney tissues were fixed in 10% neutral buffered formalin (Beijing Yili Fine Chemical Co., Ltd., Beijing, China), followed by embedding into paraffin. The paraffin-embedded tissue sections were sectioned into 4-μm-thick slices utilizing a rotary sectioning machine (Leica Biosystems, Wetzlar, Germany). These slices underwent a dewaxing process with adhesive sheets and were subsequently baked at 60 °C in an oven (Beijing Yongguangming Medical Appliance Factory, Beijing, China). The immunohistochemical procedures encompassed antigen retrieval, a blocking procedure employing normal goat serum (10% in TBST), and incubation with primary antibodies [rabbit anti-GLP1 (1:100; ABclonal, Woburn, MA, USA) and rabbit anti-NFκB2 (p100/p52) (1:100; Proteintech, Rosemont, IL, USA)]. Visualization of the results was achieved using appropriate secondary antibodies and diaminobenzidine as the chromogen. The expression levels of the corresponding antibodies in the tissues were determined under a laboratory microscope (U-HL100.3, Olympus, Tokyo, Japan) at magnifications of 20×/40×. The mean optical density values (IOD) of the positive reactions in each tissue were quantified using the Image J software for statistical evaluation.

### Statistical analysis

To examine the differences between groups, comparisons between two groups following normal distribution were performed using a two-tailed student’s *t*-test, while one-factor ANOVA was used for comparisons of three or more groups. For nonparametric distributions, the Kruskal–Wallis *H* was performed for comparisons between two groups or three or more groups, respectively. All data are presented as mean ± SD, as indicated. Statistical tests used to compare conditions are indicated in figure legends (*P < 0.05; **P < 0.01; ***P < 0.001), where *P* < 0.05 was considered significant. R version 3.4.3 (https://www.r-project.org/), Python version 2.7.6 (https://www.python.org/), SPSS 26.0 (Statistical Package for the Social Sciences Software, SPSS Inc., Chicago, IL, USA), Image J (https://sourceforge.net/projects/imagej/), and Cytoscape version 3.8.2 (https://cytoscape.org/) were used for the generation of graphs and statistics.

## Results

### Clinical baseline characteristics of the subjects

A total of 21 subjects were enrolled in this study, including seven healthy subjects as the N group, 7 patients with T2DM who had a longer course as the YYLXD group, and 7 patients with T2DM and other TCM syndromes in the NYYLXD group. Compared to the N group, FPG (*P* < 0.05), HbA1C (*P* < 0.05), and age (*P* < 0.01) were all significantly increased in the YYLXD group; FPG (*P* < 0.01) was significantly increased in the NYYLXD group. Compared to the NYYLXD group, age (*P* < 0.05) was significantly increased in the YYLXD group (Table 1).

**Table 1:** Clinical characteristics of the patients, *n* = 7; values are presented as mean ± SD

Characteristics	*N*	YYLXD	NYYLXD	*P*-value
Female/male	4/3	4/3	3/4	
Age (year)	45.14 ± 7.90	63.86 ± 6.74^**※^	53.14 ± 9.69^※^	0.000^a^, 0.084^b^, 0.025^c^
BMI (kg/m2)	24.25 ± 2.16	24.11 ± 3.92	24.53 ± 0.34	0.940^a^, 0.917^b^, 0.871^c^
FPG (mmol/L)	5.27 ± 0.65	8.22 ± 2.08^*^	10.61 ± 5.07^##^	0.012^a^, 0.004^b^, 0.718^c^
HbA1C (%)	5.73 ± 0.37	8.29 ± 1.65^*^	7.77 ± 1.70	0.016^a^, 0.052^b^, 0.542^c^
TC (mmol/L)	5.20 ± 0.70	4.57 ± 1.59	5.31 ± 1.99	0.449^a^, 0.895^b^, 0.377^c^
TG (mmol/L)	2.07 ± 1.08	2.01 ± 1.45	3.67 ± 3.99	0.967^a^, 0.252^b^, 0.236^c^
LDL-C (mmol/L)	3.02 ± 0.76	2.66 ± 1.03	2.82 ± 1.16	0.506^a^, 0.702^b^, 0.775^c^
HDL-C (mmol/L)	1.35 ± 0.65	1.18 ± 0.29	1.17 ± 0.19	0.471^a^, 0.428^b^, 0.941^c^

One-factor ANOVA was applied to continuous variables (age, body mass index (BMI), HbA1C, TC, TG, LDL-C, and HDL-C). FPG was tested by Kruskal–Wallis *H* to estimate the statistical significance of the difference between groups, *α* = 0.05

^a^*P*, *P, YYLXD vs N; *P < 0.05; **P < 0.01

^b^*P*, ^#^*P*, NYYLXD vs N; ^#^*P* < 0.05; ^##^*P* < 0.01

^c^*P*, ^※^*P*, YYLXD vs NYYLXD; ^※^*P* < 0.05.

### Tryptophan derivatives and prolonged T2DM

**Fig. 1:**
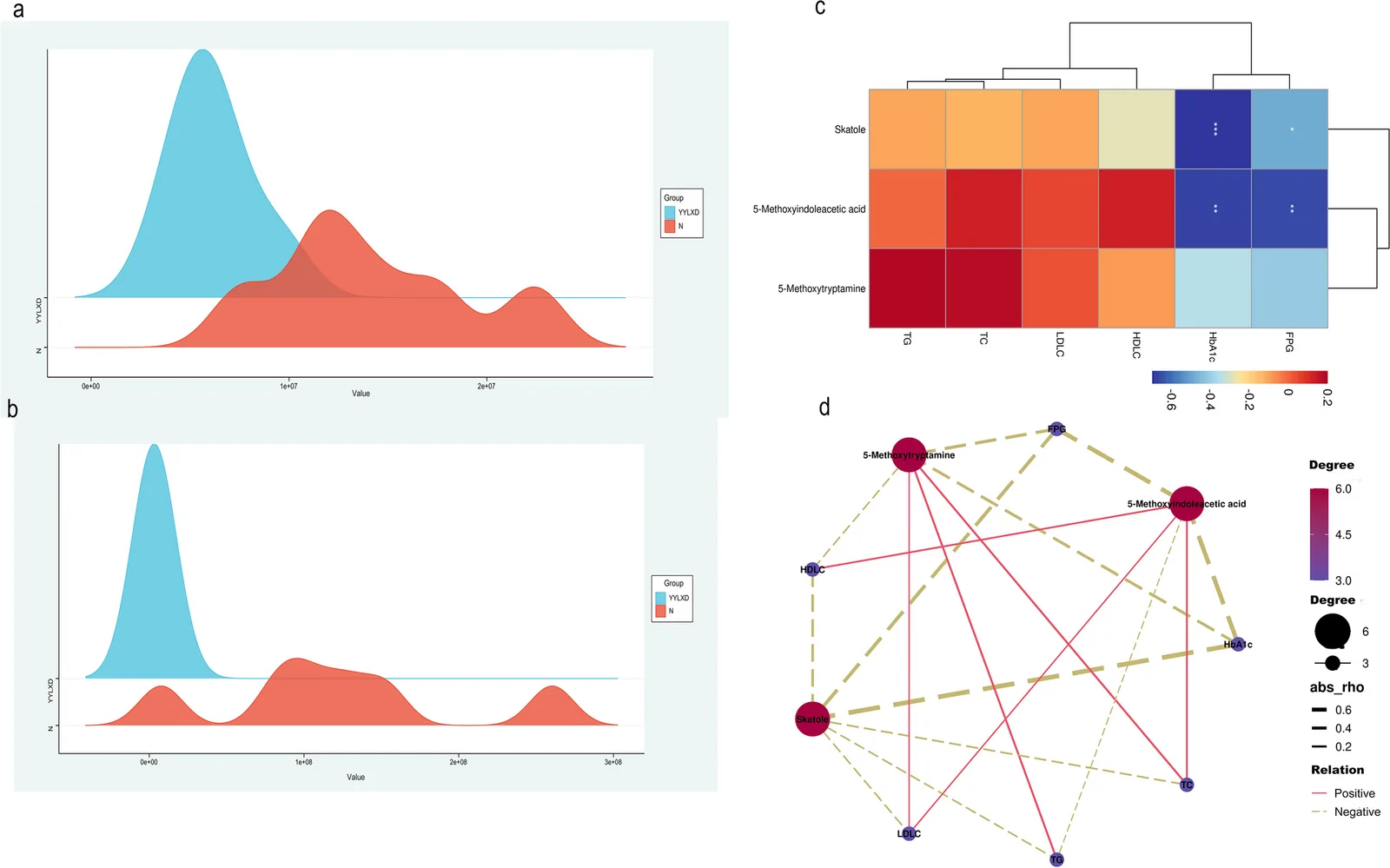
Tryptophan derivatives and prolonged T2DM **a** 5MIAA data distribution and **b** skatole data distribution between YYLXD and N groups. **c** and **d** Correlation analysis between tryptophan derivatives and clinical and biochemical indicators of T2DM. *P < 0.05;**P < 0.01 as determined by Spearman analysis. *P* < 0.05 was considered significant

Untargeted metabolomics of the subjects’ fecal samples showed that three tryptophan derivatives, skatole, 5-methoxyindoleacetic acid (5MIAA), and 5-methoxytryptamine were screened in the YYLXD versus the N comparison group, but no tryptophan derivatives were screened in the YYLXD versus NYYLXD comparator group. Compared to the normal group, skatole (*P* < 0.01), 5MIAA (*P* < 0.01) and 5-methoxytryptamine (*P* < 0.05) were all significantly decreased in the YYLXD group (Table 2). Of these, the greater downward revision of skatole was relatively (Fig. 1a and b). In addition, 5MIAA was significantly negatively correlated with both FPG (*P* < 0.01) and HbA1c (*P* < 0.01). Skatole was significantly negatively correlated with HbA1c (*P* < 0.001) and negatively correlated with FPG (*P* < 0.05) (Fig. 1c and d).

**Table 2:** Tryptophan derivative information in the YYLXD and N groups

Name	FC	log2FC	*P*-value	Up, down
5-Methoxyindoleacetic acid	0.432648198	−1.2087337	0.002060404	down
5-Methoxytryptamine	0.256043556	−1.965538846	0.021920382	down
Skatole	0.026723088	−5.225769448	0.000396416	down

### Gut microbiome and prolonged T2DM

**Fig. 2:**
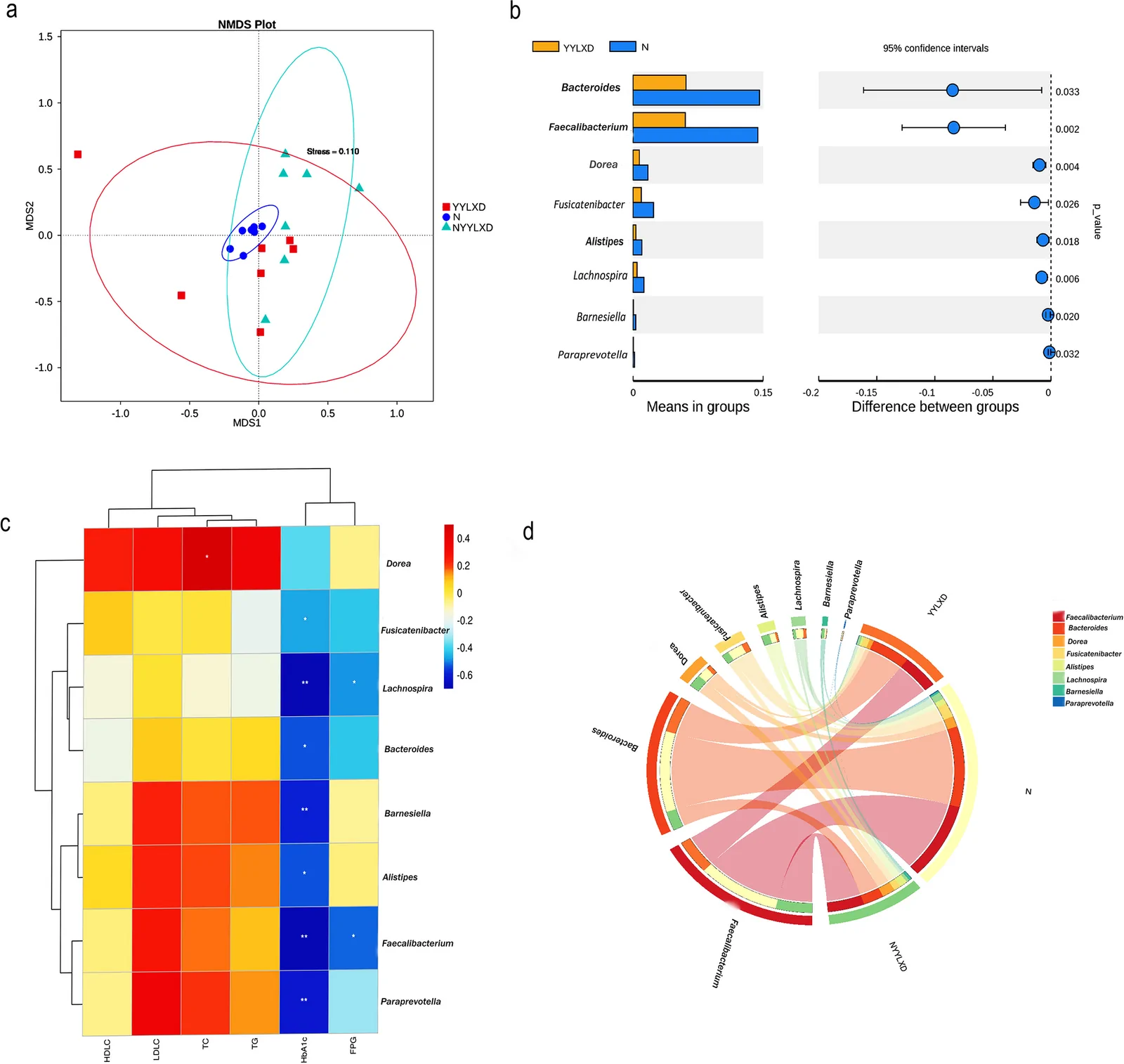
T2DM correlates with changes in the community structure of the intestinal bacterial microbiome. **a** and **c** Composition differences in gut microorganisms and correlation analysis of significantly different gut microorganisms and clinical and biochemical indicators of T2DM between YYLXD, N, and NYYLXD comparison groups at the genus level. *P < 0.05; **P < 0.01 as determined by Spearman analysis. *P* < 0.05 was considered significant. **b** Gut microorganisms with significant differences between the YYLXD and N groups at the genus level. **d** Relationship between significant gut microorganisms and the YYLXD, N, and NYYLXD groups

The results showed that there were differences in the composition of the bacterial gut microbiome between the YYLXD, N, and NYYLXD groups (Fig. 2a). Compared to the normal group, eight significantly different enteric genera (*P* < 0.05) were significantly decreased in the YYLXD group (Fig. 2b). There were no significantly different intestinal microorganisms between the YYLXD versus NYYLXD groups. In addition, *Paraprevotella* (*P* < 0.01), *Faecalibacterium* (*P* < 0.01), *Alistipes* (*P* < 0.05), *Barnesiella* (*P* < 0.01), *Bacteroides* (*P* < 0.05), *Lachnospira* (*P* < 0.01)*,* and *Fusicatenibacter* (*P* < 0.05) were all significantly negatively correlated with HbA1c; *Faecalibacterium* (*P* < 0.05) and *Lachnospira* (*P* < 0.05) were negatively correlated with FPG (Fig. 2c). All of the above gut microorganisms were correlated with N, YYLXD, and NYYLXD groups (Fig. 2d).

### Gut microbiome and tryptophan derivatives

**Fig. 3:**
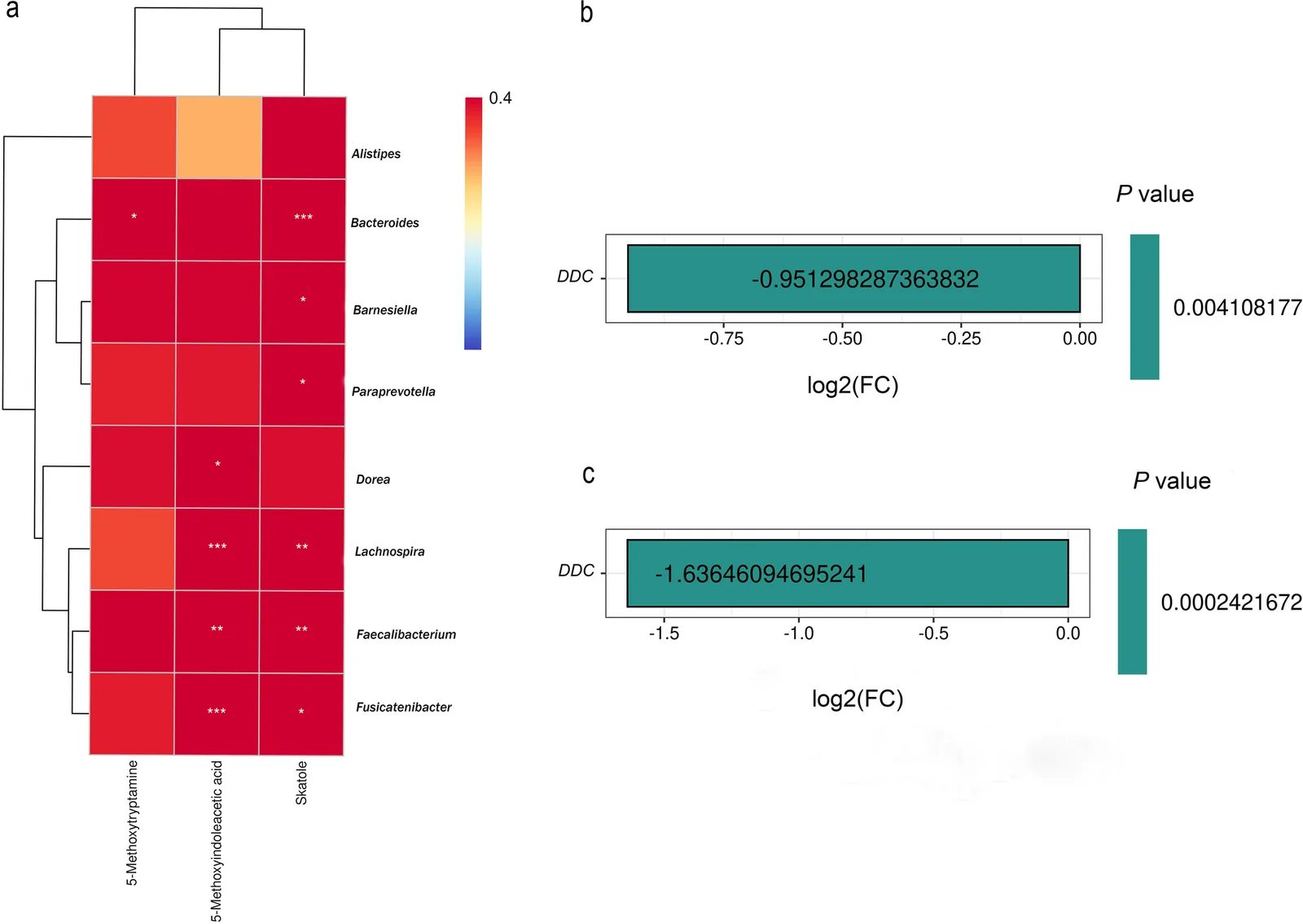
Correlation between significant gut microorganisms and tryptophan derivatives. *P < 0.05; **P < 0.01 as determined by Spearman analysis. *P* < 0.05 was considered significant. **b** The expression of *DDC* between YYLXD and N groups. **c** The expression of *DDC* between YYLXD and NYYLXD groups

To explore the relationship between significant gut microorganisms and tryptophan derivatives, we performed a correlation analysis between them. The results showed that *Fusicatenibacter* (*P* < 0.01), *Faecalibacterium* (*P* < 0.01), *Lachnospira* (*P* < 0.01), and *Dorea* (*P* < 0.05) were all positively correlated with 5MIAA; *Fusicatenibacter* (*P* < 0.05), *Faecalibacterium* (*P* < 0.01), *Lachnospira* (*P* < 0.01), *Paraprevotella* (*P* < 0.05), *Barnesiella* (*P* < 0.05), *Bacteroides* (*P* < 0.01) were all positively correlated with skatole. In addition, *Bacteroides* (*P* < 0.05) had a positive correlation with 5-methoxytryptamin (Fig. 3a). Besides, compared to the normal group, the expression of the gene for Dopa decarboxylase ( *DDC)* (*P* < 0.01) was significantly decreased in the YYLXD group (Fig. 3b ). Compared to the NYYLXD group, *DDC* (*P* < 0.01) was significantly decreased in the YYLXD group (Fig. 3c).

### RNA changes of the RLRs signaling pathway in the peripheral blood of subjects

In the present study, RNA-seq was performed on the peripheral blood samples. The results indicated that 8 significantly different mRNAs and 44 long non-coding RNAs (lncRNAs) of the RLRs signaling pathway were identified between the YYLXD group and the normal group. Among these, compared to the normal group, *TANK* (*P* < 0.05), *TANK-012* (*P* < 0.01), *RELA* (*P* < 0.05), and *RELA-005* (*P* < 0.05) were all upregulated in the YYLXD group (Table 3 and Supplemental Table S1). Additionally, a total of 23 significantly different mRNAs and 9 LncRNAs of the RLRs signaling pathway were identified between the YYLXD and NYYLXD groups. Compared to the NYYLXD group, *TANK* (*P* < 0.01) and *TANK-013* (*P* < 0.05) were upregulated in the YYLXD group (Table 4 and Supplemental Table S2). In both pairs of comparison groups, the relative variability was greater for *TANK*. *TANK* encodes the TRAF family member-associated *NF-κB* activator.

**Table 3:** mRNA with significant differences in the RLRs signaling pathway in the peripheral blood of subjects between the YYLXD and N groups

Gene_id	Gene_name	FoldChange	log2FoldChange	*P*-value	padj
ENSG00000136560	*TANK*	1.226546449	0.294601869	0.032964489	0.546522606
ENSG00000100906	*NFKBIA*	1.300294065	0.378837929	0.001152324	0.16303992
ENSG00000188130	*MAPK12*	1.307841645	0.387187868	0.035327856	0.551760716
ENSG00000003400	*CASP10*	0.842106842	−0.247924808	0.039249014	0.567530516
ENSG00000173039	*RELA*	1.174443301	0.231977066	0.048561194	0.609185538
ENSG00000232810	*TNF*	1.337849238	0.419915548	0.012968225	0.392021876
ENSG00000115267	*IFIH1*	0.662797722	−0.593359451	0.037306594	0.556990168
ENSG00000083799	*CYLD*	0.870859489	−0.199488133	0.018468672	0.454706877

**Table 4:** mRNA with a significant difference in the RLRs signaling pathway in the peripheral blood of subjects between the YYLXD and NYYLXD groups

Gene_id	Gene_name	FoldChange	log2FoldChange	*P*-value	padj
ENSG00000136560	*TANK*	1.913295597	0.936059782	1.15E-06	5.44E-05
ENSG00000145782	*ATG12*	1.56074727	0.642236942	0.000174307	0.002531647
ENSG00000127191	*TRAF2*	0.732050488	−0.449984942	0.004706114	0.032255095
ENSG00000104365	*IKBKB*	0.783092363	−0.352745616	0.025198796	0.112196789
ENSG00000188130	*MAPK12*	0.69529583	−0.524301157	0.029557529	0.126129817
ENSG00000185386	*MAPK11*	0.478318966	−1.063955098	0.000624252	0.006697651
ENSG00000003400	*CASP10*	0.780621773	−0.357304392	0.018211681	0.088794311
ENSG00000064012	*CASP8*	1.225437457	0.293296855	0.04878144	0.180347901
ENSG00000160703	*NLRX1*	0.664303931	−0.590084644	0.001494392	0.013274353
ENSG00000126456	*IRF3*	0.57992344	−0.786065643	6.06E-07	3.28E-05
ENSG00000185507	*IRF7*	0.590770401	−0.759330551	0.000887947	0.008788637
ENSG00000102871	*TRADD*	0.500047854	−0.999861929	2.68E-09	5.28E-07
ENSG00000108771	*DHX58*	0.591073384	−0.758590838	0.000316058	0.003942598
ENSG00000131323	*TRAF3*	0.658920141	−0.601824469	0.00013305	0.002044438
ENSG00000107643	*MAPK8*	1.370461031	0.454661305	0.005357618	0.035555739
ENSG00000175104	*TRAF6*	1.365766575	0.449710932	0.005805875	0.037861951
ENSG00000104825	*NFKBIB*	0.663280854	−0.592308212	0.001841307	0.015576881
ENSG00000109320	*NFKB1*	0.740014392	−0.434374766	0.002137584	0.017414806
ENSG00000107201	*DDX58*	1.412320302	0.498067317	0.010857783	0.060434053
ENSG00000127445	*PIN1*	0.686881603	−0.541866651	0.00078563	0.007993839
ENSG00000052723	*SIKE1*	1.43261579	0.518651748	0.003363754	0.024835067
ENSG00000213341	*CHUK*	1.278142337	0.354048508	0.018149863	0.088519616
ENSG00000135341	*MAP3K7*	1.375605466	0.460066754	0.003136328	0.023488663

### Correlation analysis

**Fig. 4:**
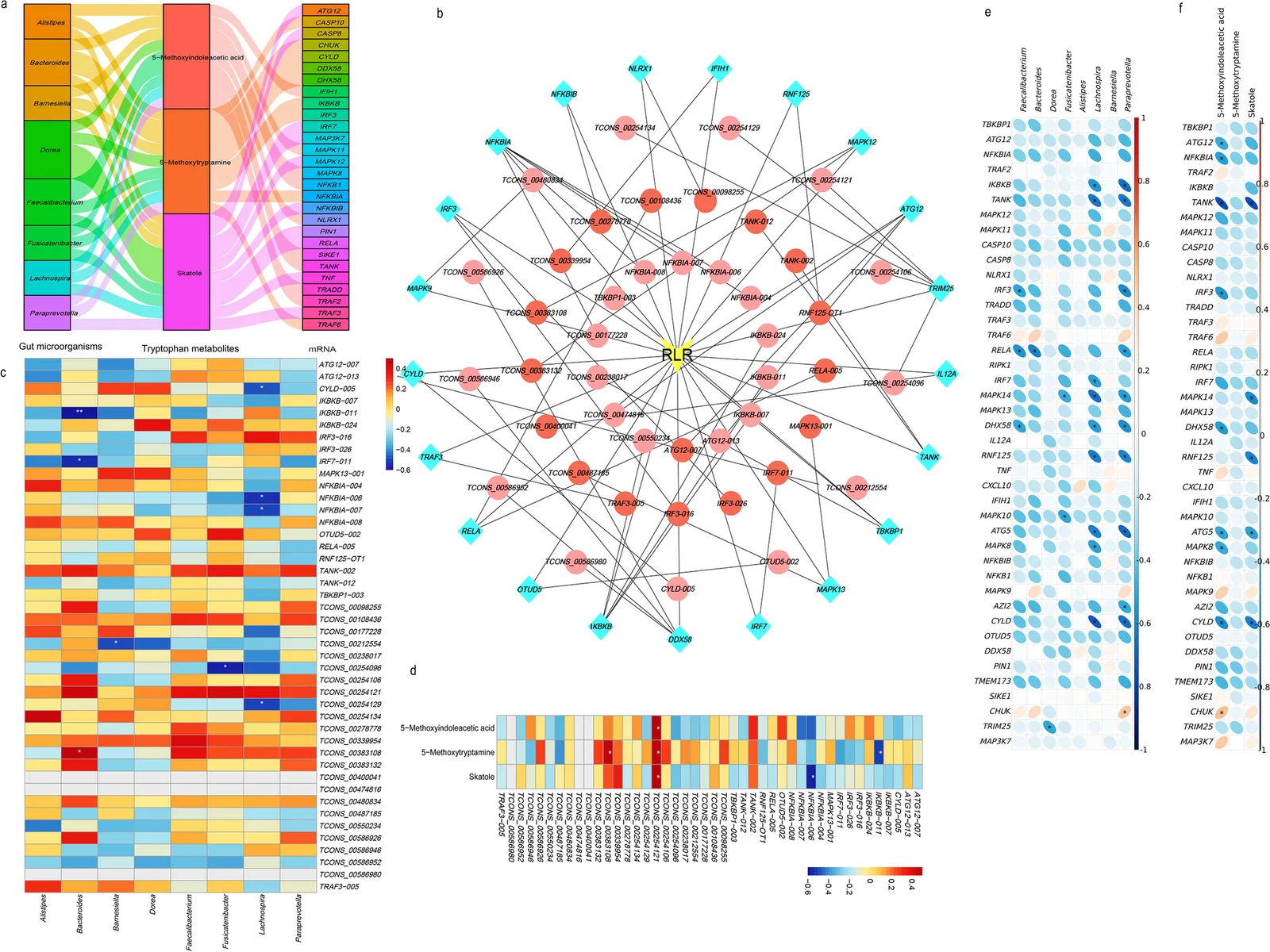
Correlation analysis. **a** Correlation analysis of significantly different gut microorganisms, tryptophan derivatives, and differential RLRs signaling pathway mRNAs. **b** Correlation analysis of differential lncRNAs and their targeted mRNAs in the RLRs signaling pathway. **c** Correlation analysis of significantly different gut microorganisms and differential lncRNAs. **d** Correlation analysis of tryptophan derivatives and differential lncRNAs. **e** Correlation analysis of significantly different gut microorganisms and significantly different RLRs signaling pathway mRNAs. **f** Correlation analysis of tryptophan derivatives and significantly different RLRs signaling pathway mRNAs. *P < 0.05; **P < 0.01 as determined by Spearman analysis. *P* < 0.05 was considered significant

To further investigate the correlation between tryptophan derivatives, significantly different gut microbes, and RNAs of the RLRs signaling pathway, a combined multi-omics analysis was performed. As lncRNAs can regulate the expression levels of target mRNAs through co-localization and co-expression, a lncRNA-mRNA relationship network was constructed. The results revealed that tryptophan derivatives and significantly different gut microbes were correlated with mRNAs of the RLRs signaling pathway (Fig. 4a), and there was a correlation between lncRNAs and their target mRNAs (Fig. 4b). Of these, 5MIAA and skatole were significantly negatively correlated with *TANK* (*P* < 0.001) and *TANK-012* (Fig. 4f and d). *Faecalibacterium* and *Bacteroides* were negatively correlated with *RELA* (*P* < 0.05) and *RELA-005*. *Lachnospira* and *Paraprevotella* were negatively correlated with *TANK* (*P* < 0.05) and *TANK-012* (Fig. 4e and c).

### Changes in pathology and inflammation-related genes in the colon of mice modeled with T2DM

**Fig. 5:**
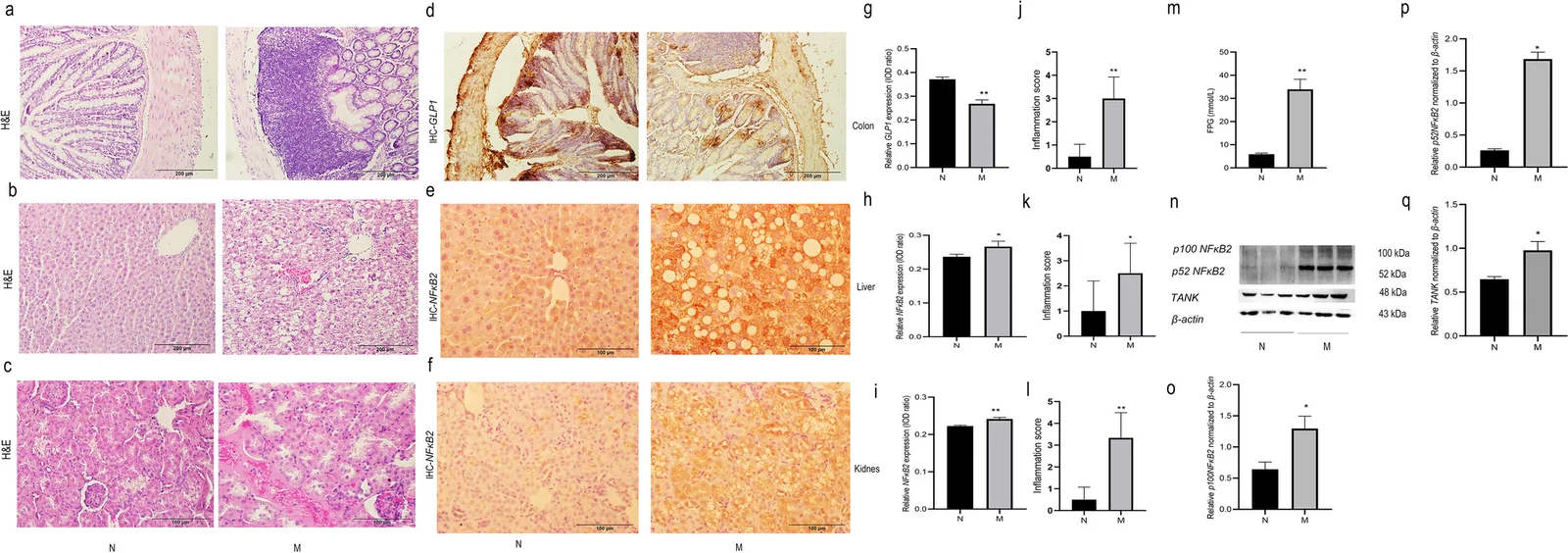
FPG value of mice, H&E staining, and expression of inflammation-related genes and GLP1 between N and M groups. H&E staining and inflammation score of **a** and **j** colon, **b** and **k** liver, and **c** and **l** kidney tissues in N and M groups. **m** FPG values of mice. **d** and **g** Expression of *GLP1* in the colon between N and M groups. Expression of *NFκB2* in **e** and **h** liver and **f** and **i** kidney tissues of mice. **n**–**q** Expressions of *p100NFκB2*, *p52NFκB2*, and *TANK* in the colon between N and M groups.*P < 0.05; **P < 0.01 as determined by Kruskal–Wallis *H* and one-factor ANOVA. *P* < 0.05 was considered significant

Subsequently, an animal model of prolonged T2DM was established. The results showed that compared to the N group, FPG (*P* < 0.01) was significantly increased in the model (M) group (*P* < 0.01), and the FPG value of the model group was more than 30.00 mmol/l (Fig. 5m). In addition, the colonic intestinal glands were neatly arranged without wrinkles, and there was no obvious inflammatory cell infiltration in the N group. Compared with mice in the N group, mice in the prolonged T2DM group exhibited abnormalities in the gut wall and intestinal glandular folds. Additionally, there was a significant presence of infiltrating inflammatory cells (Fig. 5a). Compared with the normal group, the inflammation scores (*P* < 0.01) of colonic tissues of mice were significantly increased in the model group (Fig. 5j). In addition, compared with the normal group, the expression of *TANK* (*P* < 0.05), *p100NFκB2* (*P* < 0.05) and *p52NFκB2* (*P* < 0.05) were all increased in the colonic tissues of mice in the M group ( Fig. 5n–q) while the expression of *GLP1* (*P* < 0.01) was significantly decreased (Fig. 5d).

### Changes in liver and kidney pathology and inflammation-related genes in mice modeled with a longer course of T2DM

We performed H&E staining on the liver and kidney of mice and examined the expression of *NFκB2* in murine liver and kidney using IHC. The result showed that in the N group, the liver tissue exhibited an intact lobular structure, with hepatocytes arranged radially around the central vein and centrally located nuclei. However, versus the N group, mice in the M group displayed recognizable hepatic lobules, while with disorganized hepatic cords. The central vein was congested with blood, and some of the cell nuclei were displaced to one side (Fig. 5b). Compared with the normal group, the inflammation scores (*P* < 0.05 ) of liver tissues were significantly increased in the model group (Fig. 5k); Regarding renal structures, the renal glomeruli and tubules of the normal mice were regularly shaped, with the epithelial cells of the tubules neatly arranged and intact, and homogeneous cytoplasm. In contrast, in the M group versus the N group, the glomeruli and tubules were congested with blood. Additionally, the tubular epithelial cells were swollen and disorganized (Fig. 5c). Meanwhile, compared with the normal group, the inflammation scores (*P* < 0.01) of kidney tissues of mice were significantly increased in the model group (Fig. 5l). In addition, compared with the normal group, the expression of *NFκB2 (p100/p52)* (*P* < 0.01) was increased in both kidney and liver tissues in the model group (Fig. 5i and h). The transfer of *p52 NFκB2* to the nucleus was observed in both liver and kidney tissues (Fig. 5e and f).

### Gut microbiome of murine

**Fig. 6:**
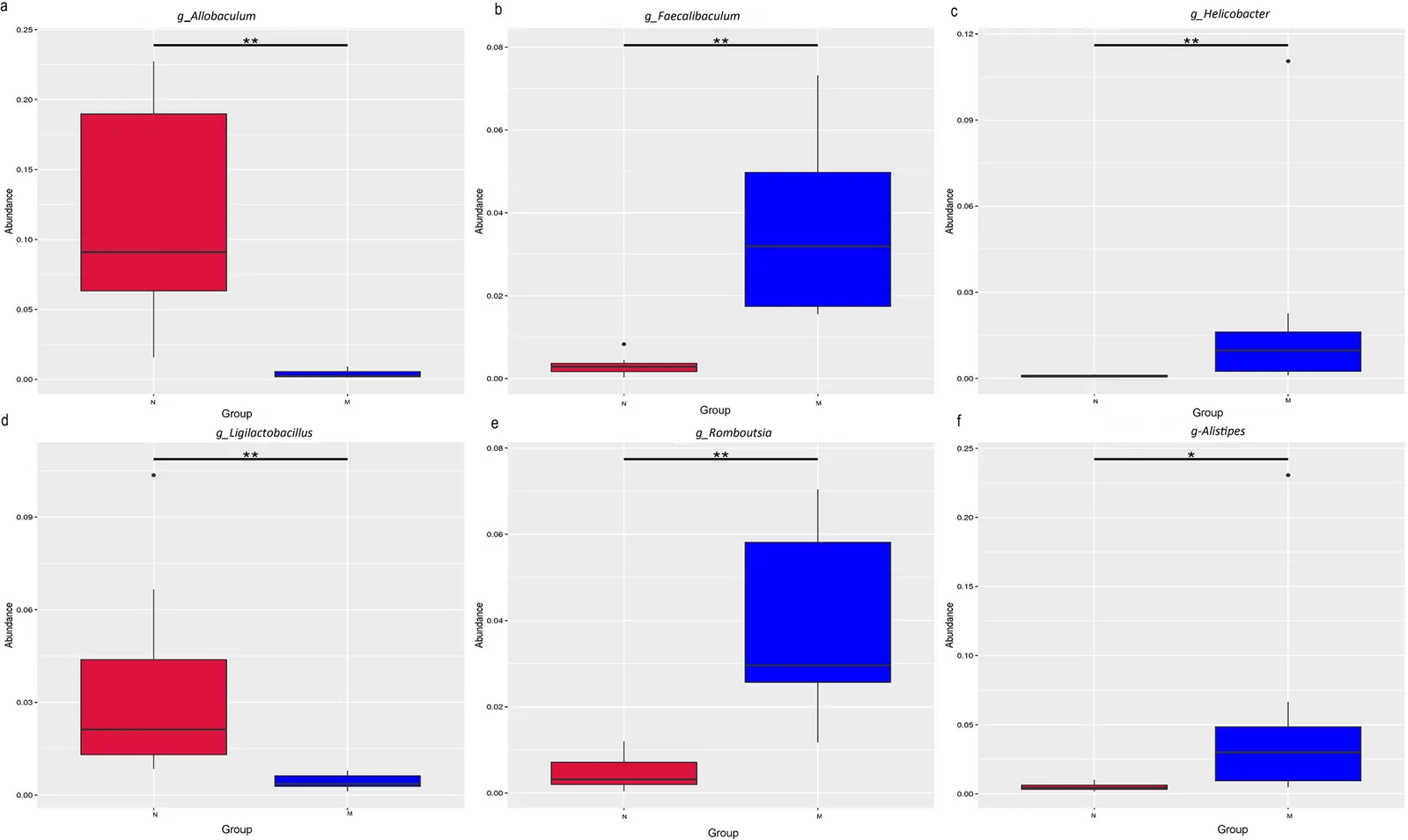
Gut microorganisms with significant differences between N and M groups (genus level). The abundance of **a** *Allobaculum*, **b** *Faecalibaculum*, **c** *Helicobacter*, **d** *Ligilactobacillus*, **e** *Romboutsia*, and **f** *Alistipes* between N and M groups.*P < 0.05; **P < 0.01 as determined by MetaStat. *P* < 0.05 was considered significant

To verify the relationship between inflammatory response and intestinal microbiome and its structural changes at the animal level, 16S rRNA gene amplification sequencing was conducted on animal feces. The results showed that there were six intestinal microorganisms with significant differences between the N and the M groups. Compared with the normal group, *Allobaculum* (*P* < 0.01) and *Ligilactobacillus* (*P* < 0.01) were all significantly decreased in the M group; *Faecalibaculum* (*P* < 0.01), *Helicobacter* (*P* < 0.01), *Romboutsia* (*P* < 0.01), and *Alistipes* (*P* < 0.05) were all significantly increased (*P* < 0.01) ( Fig. 6).

## Discussion

Gut microbes, such as *Bacteroid*es can metabolize tryptophan to produce indole-3-acetic acid (IAA) (Smith and Macfarlane 1997) and then convert IAA to skatole by coding for enzyme DDC. Studies demonstrated that *Bacteroides* have positive influences on glucose metabolism (Gauffin Cano et al. 2012; Yang et al. 2017), and they can regulate glycan synthesis (Yan et al. 2022). Besides, *Bacteroides* may serve as one of the intestinal microorganisms that catabolize tryptophan to produce indole (Keszthelyi et al. 2009) that can stimulate the production of GLP1 by intestinal endocrine L cells, which in turn stimulates insulin secretion by pancreatic β cells and exerts hypoglycemic effect (Zhang et al. 2021). The enzyme DDC is an integral component of skatole metabolism (Ouwerkerk et al. 1999; Whitehead et al. 2008). The glucose concentration in the intestine significantly affects the activity of DDC, in turn affecting skatole production (Yokoyama and Carlson 1979). Nevertheless, an ample presence of skatole is crucial for the establishment of the microbial-indole axis (Hubbard et al. 2015).

In the present study, it was found that the proportions of *Bacteroides*, *DDC*, and skatole were significantly reduced in the YYLXD group compared with those in the N group. In addition, *Bacteroides* and skatole were negatively correlated with HbA1c levels. Furthermore, decreased GLP1 expression levels in the colon tissue of mice simulated the longer course of T2DM. Prior research has demonstrated that T2DM could lead to dysbiosis (Gutiérrez-Salmerón et al. 2021). This suggests that suppression of DDC activity and decrease of *Bacteroides* abundance may be associated with the risk of T2DM, which has a longer course and results in the reduction of skatole production. Moreover, a low abundance of *Bacteroides* or reduced skatole production may further lead to increased blood glucose levels by downregulating the *GLP1* expression level.

In addition, Kang et al. (2019) found that the abundance of *Alistipes* was positively correlated with TC and TG levels in the blood (Kang et al. 2019). Previous studies have shown that *Alistipes* could indirectly activate gluconeogenesis by producing butyrate to participate in the process of glucose metabolism (Vadder et al. 2014; Wu et al. 2022). Moreover, scholars have demonstrated that diabetes and blood lipids exhibit a correlation, where TC and TG are integral components of blood lipids (Jia et al. 2021). An elevated TG level contributes to the increased IR, leading to reduced glucose uptake and elevated blood glucose level (Blüher et al. 2001; Dresner et al. 1999). Additionally, IR can further elevate TG levels (Boden 1997). Studies have indicated that the abundance of butyrate-producing bacteria decreases in diabetic mice with lipid metabolism disorders (Qin et al. 2012; Wang et al. 2020). The findings of the present study revealed a decreased abundance of *Alistipes* in the YYLXD group compared with the N group. Notable, TG and TC levels in the YYLXD group did not significantly differ from those in the control group. Thus, the results of the present study, associated with findings from other researchers, underscore the potential regulatory role of gut microbes in host lipid metabolism (Chen et al. 2019; Fu et al. 2015).

*Bacteroides* and *Faecalibacterium* can attenuate the inflammatory response by regulating RELA (Kelly et al. 2004; Zhu et al. 2021), and *Faecalibacterium* abundance is negatively correlated with the levels of inflammatory cytokines. Previous studies have concluded that *RELA* is a member of the NF-κB family, increasing the subsequent production of large amounts of pro-inflammatory cytokines (Karunakaran et al. 2021). Moreover, *RELA* is one of the RLRs signaling pathway-associated genes (Ding et al. 2017). The results of the present study indicated that *Bacteroides* and *Faecalibacterium* were negatively correlated with *RELA*. Besides, *RELA* expression level was upregulated, although the abundances of *Bacteroides* and *Faecalibacterium* were reduced in the YYLXD group. Therefore, these findings suggest that in prolonged T2DM, low abundances of *Bacteroides* and *Faecalibacterium* may trigger the RLRs signaling pathway by upregulating RELA expression level, which subsequently induces the release of inflammatory cytokines.

To further clarify the relationship among the RLRs signaling pathway, gut microbiota, immune dysfunction, and prolonged T2DM, an animal model of T2DM was established by intraperitoneal injection of STZ. STZ-induced T2DM is a widely accepted model for the assessment of the pathobiology of T2DM (Bathina and Das 2018). To conform to the clinicopathological course of T2DM with complications (e.g., fatigue and frailty, nocturnal polyuria, soreness, weakness of the waist and knees, dizziness and discomfort, and thin stools), the extended modeling time displayed symptoms, such as emaciation, huddling, slow movement, and lackluster body hair. Research indicated that inflammation can impact the kynurenine pathway of tryptophan metabolism, inducing significant alterations in the indolamine-2,3-dioxygenase enzyme and influencing the production of neuroactive metabolites (Widner et al. 1997). This process plays a crucial role in the development of neuropsychiatric symptoms, including fatigue and dizziness (Kim et al. 2015). Furthermore, the characteristics of physical frailty, weakness, and slowness are regulated by neuromuscular action (Westbrook et al. 2020). Gonzalez-Freire et al. (2014) discovered that chronic inflammation could lead to the decline of neuromuscular function, encompassing spinal neurons, peripheral axons, and muscle tissue (Gonzalez-Freire et al. 2014). The elevated NF-κB expression level can induce muscle and bone weakness. Additionally, skatole has the potential to inhibit inflammatory reactions by suppressing lipid oxidation (Adams et al. 1987; Liu et al. 2020). It can be inferred that the physical frailty and weakness of the waist and knees may be associated with the decline of neuromuscular function induced by the low levels of skatole.

Nocturnal polyuria is one of the main clinical symptoms of T2DM patients with a longer course (Guo et al. 2014). As the kidney is an important excretory organ, the metabolites produced by intestinal microorganisms may first enter the liver via the portal circulation system that may influence the whole organism through the capillary network, and skatole can affect the liver homeostasis (Saeedi et al. 2020). The expression level of *NF-κB* in murine kidneys was identified in the present study to clarify whether the phenomenon of nocturnal polyuria could be associated with the activation of the NF-κB signaling pathway by low levels of probiotics, causing inflammatory damage to the kidneys. Additionally, the expression levels of inflammation-related genes in the murine colon and liver tissues were detected to determine whether the NF-κB signaling pathway, which was triggered by the low skatole production, could cause inflammatory damage to the liver. The results revealed that upregulation of *TANK* expression level in the animal’s colon tissue (consistent with RNA-seq results in human peripheral blood samples) activated NFκB2, resulting in inflammatory damage to the liver, kidney, and colon. *TANK* is a member of the TRAF family that is associated with the NF-κB activator (Huh et al. 2020), and *TANK* is one of the RLR signaling pathway-associated genes (Chen and Jiang 2013; Eisenächer and Krug 2012). Both NFκB2 and RELA are members of the NF-κB family. Chawla et al. (2021) found that the NFκB2/RELA complex is present in intestinal epithelial cells (Chawla et al. 2021). *RELA* becomes activated, subsequently triggering NFκB2 (Ghosh and Wang 2021). In addition, it was demonstrated that NFκB2 expression level was significantly elevated in aged tissues (Bernal et al. 2014 ). Moreover, previous studies have reported that exposure to STZ alone did not activate the NF-κB signaling pathway (Wright et al. 2021). It is evident that the activation of the NFκB2 signaling observed in this study is associated with the presence of T2DM itself.

Besides, the present study revealed that dysbiosis was identified in the intestinal microbiome of mice in the M group and the intestinal microbiome of T2DM patients with a longer course, representing a significant reduction in the abundance of probiotics. Studies have demonstrated that dysbiosis is closely associated with inflammation and metabolic syndrome (Sen et al. 2017). The gut microorganisms in the normal murine tissue were mainly *Allobaculum* and *Ligilactobacillus*, and the abundances of *Allobaculum* and *Ligilactobacillus* were significantly reduced in the M group compared with those in the N group. It was reported that *Allobaculum* and *Ligilactobacillus* could produce butyric acid that has anti-inflammatory effects (Andoh et al. 1999; Yao et al. 2021; Zheng et al. 2021). On the other hand, the main human intestinal microorganisms (*Bacteroides* and *Faecalibacterium*) can also exert anti-inflammatory effects by producing butyric acid (Deb et al. 2020). It suggests that a decrease in the abundance of butyric acid-producing bacteria may induce inflammation in the longer pathological T2DM process. In addition, the abundance of pathogenic bacteria *Helicobacter* in the intestinal microbiome of mice in the M group was significantly elevated compared with that in the N group. Prior research has indicated that *Helicobacter pylori* could promote inflammation and induce indoleamine 2,3-dioxygenase 1 (IDO-1) activity (Feng et al. 2020). IDO-1 is the first rate-limiting enzyme that is responsible for the degradation of tryptophan (Barat et al. 2016). *Bacteroides* can exert inflammatory effects by encoding DDC to metabolize tryptophan into skatole (Smith and Macfarlane 1997). It can be concluded that in the longer pathological T2DM process, although the compositions of animal intestinal microorganisms and human intestinal microorganisms are different, they may eventually lead to dysbiosis, which is attributable to inflammation and tryptophan metabolism. Thus, the present study indicated that the RLRs signaling pathway may be triggered by intestinal flora and may further activate its downstream NF-κB signaling pathway, inducing an inflammatory response in T2DM with a longer course.

In conclusion, this study presented a fresh perspective on the intricate relationship among the RLRs signaling pathway, gut microbiota, microbial tryptophan derivatives, inflammation, and the prolonged course of T2DM. On one side, hyperglycemia could potentially suppress tryptophanase activity, and the extended duration of T2DM might reduce probiotic abundance. Conversely, the decreased abundance of *Bacteroides*, along with reduced DDC enzymatic activity, could result in a decline in skatole production. This reduction in *Bacteroides* or skatole levels could contribute to the elevated blood glucose level by decreasing GLP1 expression level. Moreover, the intestinal microbiome could act as a regulator of lipid metabolism. Additionally, a diminished abundance of probiotics could trigger an inflammatory response through the RLRs signaling pathway, subsequently activating the downstream NF-κB signaling pathway in prolonged T2DM. Throughout this process, lncRNAs, such as *TANK-012* and *RELA-005*, could play a crucial role. These findings hold promise for the targeted use of specific microbes or microbially derived small molecules for treating T2DM with an extended course. However, certain limitations of this study should be noted, such as the small sample size and the absence of mechanistic insights, which will be addressed in future research, including experiments involving genetically modified animals and fecal microbiota transplantation in animal models of T2DM.

## Supplementary information

ESM 1

## Data Availability

BioSample accession numbers (National Center for Biotechnology Information (NCBI)) of 16S rRNA gene amplicon sequencing: SAMN30805922; SAMN30805923; SAMN30805924; SAMN30805925; SAMN30805926; SAMN30805927; SAMN30805928; SAMN30805929; SAMN30805930; SAMN30805931; SAMN30805932; SAMN30805933; SAMN30805934; SAMN30805935; SAMN30805936; SAMN30805936; SAMN30805936; SAMN30805937; SAMN30805938; SAMN30805939; SAMN30805940; SAMN30805941; SAMN30805942. BioSample accession number (NCBI) of RNA-seq is PRJNA982291.

## References

[CR1] Adams JD, Heins MC, Yost GS (1987) 3-Methylindole inhibits lipid peroxidation. Biochem Biophys Res Commun 149(1):73–78. 10.1016/0006-291x(87)91606-8 3689418 10.1016/0006-291x(87)91606-8

[CR2] Andoh A, Bamba T, Sasaki M (1999) Physiological and anti-inflammatory roles of dietary fiber and butyrate in intestinal functions. JPEN J Parenter Enteral Nutr 23(5 Suppl):S70–S73. 10.1177/014860719902300518 10483900 10.1177/014860719902300518

[CR3] Barat P, Meiffred M-C, Brossaud J, Fuchs D, Corcuff J-B, Thibault H, Capuron L (2016) Inflammatory, endocrine and metabolic correlates of fatigue in obese children. Psychoneuroendocrinology 74:158–163. 10.1016/j.psyneuen.2016.09.002 27627133 10.1016/j.psyneuen.2016.09.002

[CR4] Bathina S, Das UN (2018) Dysregulation of *PI3K-Akt-mTOR* pathway in the brain of streptozotocin-induced type 2 diabetes mellitus in Wistar rats. Lipids Health Dis 17(1):168. 10.1186/s12944-018-0809-2 30041644 10.1186/s12944-018-0809-2 PMC6058366

[CR5] Bernal GM, Wahlstrom JS, Crawley CD, Cahill KE, Pytel P, Liang H, Kang S, Weichselbaum RR, Yamini B (2014) Loss of *NFKB1* leads to early onset aging. Aging 6(11):931–943. 10.18632/aging.100702 25553648 10.18632/aging.100702 PMC4276787

[CR6] Blüher M, Kratzsch J, Paschke R (2001) Plasma levels of tumor necrosis factor-alpha, angiotensin II, growth hormone, and *IGF-I* are not elevated in insulin-resistant obese individuals with impaired glucose tolerance. Diabetes care 24(2):328–334. 10.2337/diacare.24.2.328 11213887 10.2337/diacare.24.2.328

[CR7] Boden G (1997) Role of fatty acids in the pathogenesis of insulin resistance and NIDDM. Diabetes 46(1):3–10 8971073

[CR8] Bulgarelli D, Garrido-Oter R, Münch PC, Weiman A, Dröge J, Pan Y, McHardy AC, Schulze-Lefert P (2015) Structure and function of the bacterial root microbiota in wild and domesticated barley. Cell Host Microbe 17(3):392–403. 10.1016/j.chom.2015.01.011 25732064 10.1016/j.chom.2015.01.011 PMC4362959

[CR9] Cervantes-Barragan L, Chai JN, Tianero MD, Di Luccia B, Ahern PP, Merriman J, Cortez VS, Caparon MG, Donia MS, Gilfillan S, Cella M, Gordon JI, Hsieh C-S, Colonna M (2017) *Lactobacillus reuteri* induces gut intraepithelial CD4+CD8αα+ T cells. Science 357(6353):806–810. 10.1126/science.aah5825 28775213 10.1126/science.aah5825 PMC5687812

[CR10] Chawla M, Mukherjee T, Deka A, Chatterjee B, Sarkar UA, Singh AK, Kedia S, Lum J, Dhillon MK, Banoth B, Biswas SK, Ahuja V, Basak S (2021) An epithelial *NFκB2* pathway exacerbates intestinal inflammation by supplementing latent *RELA* dimers to the canonical *NF-κB* module. Proc Natl Acad Sci U S A 118(25). 10.1073/pnas.2024828118 10.1073/pnas.2024828118 PMC8237674 34155144

[CR11] Chen H, Jiang Z (2013) The essential adaptors of innate immune signaling. Protein Cell 4(1):27–39. 10.1007/s13238-012-2063-0 22996173 10.1007/s13238-012-2063-0 PMC4875439

[CR12] Chen J-J, Xie J, Zeng B-H, Li W-W, Bai S-J, Zhou C, Chen W, Wei H, Xie P (2019) Absence of gut microbiota affects lipid metabolism in the prefrontal cortex of mice. Neurol Res 41(12):1104–1112. 10.1080/01616412.2019.1675021 31587617 10.1080/01616412.2019.1675021

[CR13] Cheng W, Shivshankar P, Li Z, Chen L, Yeh I-T, Zhong G (2008) *Caspase-1* contributes to chlamydia trachomatis-induced upper urogenital tract inflammatory pathologies without affecting the course of infection. Infect Immun 76(2):515–522. 10.1128/IAI.01064-07 18025098 10.1128/IAI.01064-07 PMC2223466

[CR14] Deb D, Das S, Adak A, Khan MR (2020) Traditional rice beer depletes butyric acid-producing gut bacteria Faecalibacterium and Roseburia along with fecal butyrate levels in the ethnic groups of Northeast India. 3 Biotech 10(6):283. 10.1007/s13205-020-02280-8 32550102 10.1007/s13205-020-02280-8 PMC7266887

[CR15] Ding Z, An K, Xie L, Wu W, Zhang R, Wang D, Fang Y, Chen H, Xiao S, Fang L (2017) Transmissible gastroenteritis virus infection induces *NF-κB* activation through RLR-mediated signaling. Virology 507:170–178. 10.1016/j.virol.2017.04.024 28448848 10.1016/j.virol.2017.04.024 PMC7111708

[CR16] Dresner A, Laurent D, Marcucci M, Griffin ME, Dufour S, Cline GW, Slezak LA, Andersen DK, Hundal RS, Rothman DL, Petersen KF, Shulman GI (1999) Effects of free fatty acids on glucose transport and *IRS-1*-associated phosphatidylinositol 3-kinase activity. J Clin Invest 103(2):253–259. 10.1172/JCI5001 9916137 10.1172/JCI5001 PMC407880

[CR17] Dyer AH, McKenna L, Batten I, Jones K, Widdowson M, Dunne J, Conlon N, Reilly R, Woods CP, O'Neill D, Gibney J, Bourke NM, Kennelly SP (2020) Peripheral inflammation and cognitive performance in middle-aged adults with and without type 2 diabetes: results from the NBIND study. Front Aging Neurosci 12:605878. 10.3389/fnagi.2020.605878 33424582 10.3389/fnagi.2020.605878 PMC7793991

[CR18] Eisenächer K, Krug A (2012) Regulation of RLR-mediated innate immune signaling--it is all about keeping the balance. Eur J Cell Biol 91(1):36–47. 10.1016/j.ejcb.2011.01.011 21481967 10.1016/j.ejcb.2011.01.011

[CR19] Fan Y, Pedersen O (2021) Gut microbiota in human metabolic health and disease. Nat Rev Microbiol 19(1):55–71. 10.1038/s41579-020-0433-9 32887946 10.1038/s41579-020-0433-9

[CR20] Feng G-J, Chen Y, Li K (2020) *Helicobacter pylori* promote inflammation and host defense through the *cagA*-dependent activation of *mTORC1*. J Cell Physiol 235(12):10094–10108. 10.1002/jcp.29826 32722876 10.1002/jcp.29826

[CR21] Fu J, Bonder MJ, Cenit MC, Tigchelaar EF, Maatman A, Dekens JAM, Brandsma E, Marczynska J, Imhann F, Weersma RK, Franke L, Poon TW, Xavier RJ, Gevers D, Hofker MH, Wijmenga C, Zhernakova A (2015) The gut microbiome contributes to a substantial proportion of the variation in blood lipids. Circ Res 117(9):817–824. 10.1161/CIRCRESAHA.115.306807 26358192 10.1161/CIRCRESAHA.115.306807 PMC4596485

[CR22] Gao J, Xu K, Liu H, Liu G, Bai M, Peng C, Li T, Yin Y (2018) Impact of the gut microbiota on intestinal immunity mediated by tryptophan metabolism. Front Cell Infect Microbiol 8:13. 10.3389/fcimb.2018.00013 29468141 10.3389/fcimb.2018.00013 PMC5808205

[CR23] Gauffin Cano P, Santacruz A, Moya SY (2012) *Bacteroides uniformis* CECT 7771 ameliorates metabolic and immunological dysfunction in mice with high-fat-diet induced obesity. PLoS One 7(7):e41079. 10.1371/journal.pone.0041079 22844426 10.1371/journal.pone.0041079 PMC3406031

[CR24] Ghosh G, Wang VY-F (2021) Origin of the functional distinctiveness of *NF-κB/p52*. Front Cell Dev Biol 9:764164. 10.3389/fcell.2021.764164 34888310 10.3389/fcell.2021.764164 PMC8650618

[CR25] Gonzalez-Freire M, Cabo R, Studenski SA, Ferrucci L (2014) The neuromuscular junction: aging at the crossroad between nerves and muscle. Front Aging Neurosci 6:208. 10.3389/fnagi.2014.00208 25157231 10.3389/fnagi.2014.00208 PMC4127816

[CR26] Guo J, Chen H, Song J, Wang J, Zhao L, Tong X (2014) Syndrome differentiation of diabetes by the traditional Chinese medicine according to evidence-based medicine and expert consensus opinion. Evid Based Complement Alternat Med 2014:492193. 10.1155/2014/492193 25132859 10.1155/2014/492193 PMC4123514

[CR27] Gutiérrez-Salmerón M, Lucena SR, Chocarro-Calvo A, García-Martínez JM, Martín Orozco RM, García-Jiménez C (2021) Remodelling of colorectal cancer cell signalling by microbiota and immunity in diabetes. Endocr Relat Cancer 28(6):R173–R190. 10.1530/ERC-20-0315 33852432 10.1530/ERC-20-0315

[CR28] Hashimoto T, Perlot T, Rehman A, Trichereau J, Ishiguro H, Paolino M, Sigl V, Hanada T, Hanada R, Lipinski S, Wild B, Camargo SMR, Singer D, Richter A, Kuba K, Fukamizu A, Schreiber S, Clevers H, Verrey F et al (2012) *Ace2* links amino acid malnutrition to microbial ecology and intestinal inflammation. Nature 487(7408):477–481. 10.1038/nature11228 22837003 10.1038/nature11228 PMC7095315

[CR29] Hess M, Sczyrba A, Egan R, Kim T-W, Chokhawala H, Schroth G, Luo S, Clark DS, Chen F, Zhang T, Mackie RI, Pennacchio LA, Tringe SG, Visel A, Woyke T, Wang Z, Rubin EM (2011) Metagenomic discovery of biomass-degrading genes and genomes from cow rumen. Science 331(6016):463–467 21273488 10.1126/science.1200387

[CR30] Hubbard TD, Murray IA, Bisson WH, Lahoti TS, Gowda K, Amin SG, Patterson AD, Perdew GH (2015) Adaptation of the human aryl hydrocarbon receptor to sense microbiota-derived indoles. Sci Rep 5:12689. 10.1038/srep12689 26235394 10.1038/srep12689 PMC4522678

[CR31] Huh JY, Reilly SM, Abu-Odeh M, Murphy AN, Mahata SK, Zhang J, Cho Y, Seo JB, Hung C-W, Green CR, Metallo CM, Saltiel AR (2020) Tank-Binding Kinase 1 regulates the localization of acyl-CoA synthetase *ACSL1* to control hepatic fatty acid oxidation. Cell Metab 32(6):1012–1027.e7. 10.1016/j.cmet.2020.10.010 33152322 10.1016/j.cmet.2020.10.010 PMC7710607

[CR32] Jia X, Xu W, Zhang L, Li X, Wang R, Wu S (2021) Impact of gut microbiota and microbiota-related metabolites on hyperlipidemia. Front Cell Infect Microbiol 11:634780. 10.3389/fcimb.2021.634780 34490132 10.3389/fcimb.2021.634780 PMC8417472

[CR33] Kang Y, Li Y, Du Y, Guo L, Chen M, Huang X, Yang F, Hong J, Kong X (2019) Konjaku flour reduces obesity in mice by modulating the composition of the gut microbiota. Int J Obes 43(8):1631–1643. 10.1038/s41366-018-0187-x 10.1038/s41366-018-0187-x 30242233

[CR34] Karunakaran D, Nguyen M-A, Geoffrion M, Vreeken D, Lister Z, Cheng HS, Otte N, Essebier P, Wyatt H, Kandiah JW, Jung R, Alenghat FJ, Mompeon A, Lee R, Pan C, Gordon E, Rasheed A, Lusis AJ, Liu P et al (2021) *Ripk1* expression associates with inflammation in early atherosclerosis in humans and can be therapeutically silenced to reduce *NF-κB* activation and atherogenesis in mice. Circulation 143(2):163–177. 10.1161/CIRCULATIONAHA.118.038379 33222501 10.1161/CIRCULATIONAHA.118.038379

[CR35] Kelly D, Campbell JI, King TP, Grant G, Jansson EA, Coutts AGP, Pettersson S, Conway S (2004) Commensal anaerobic gut bacteria attenuate inflammation by regulating nuclear-cytoplasmic shuttling of *PPAR-gamma* and *RELA*. Nat Immunol 5(1):104–112. 10.1038/ni1018 14691478 10.1038/ni1018

[CR36] Kerner W, Brückel J (2014) Definition, classification and diagnosis of diabetes mellitus. Exp Clin Endocrinol Diabetes 122(7):384–386. 10.1055/s-0034-1366278 25014088 10.1055/s-0034-1366278

[CR37] Keszthelyi D, Troost FJ, Masclee AAM (2009) Understanding the role of tryptophan and serotonin metabolism in gastrointestinal function. Neurogastroenterol Motil 21(12):1239–1249. 10.1111/j.1365-2982.2009.01370.x 19650771 10.1111/j.1365-2982.2009.01370.x

[CR38] Khosla S, Samakkarnthai P, Monroe DG, Farr JN (2021) Update on the pathogenesis and treatment of skeletal fragility in type 2 diabetes mellitus. Nat Rev Endocrinol 17(11):685–697. 10.1038/s41574-021-00555-5 34518671 10.1038/s41574-021-00555-5 PMC8605611

[CR39] Kim S, Miller BJ, Stefanek ME, Miller AH (2015) Inflammation-induced activation of the indoleamine 2,3-dioxygenase pathway: relevance to cancer-related fatigue. Cancer 121(13):2129–2136. 10.1002/cncr.29302 25728366 10.1002/cncr.29302

[CR40] Liu H, Gambino F, Algenio CS, Wu C, Gao Y, Bouchard CS, Qiao L, Bu P, Zhao S (2020) Inflammation and oxidative stress induced by lipid peroxidation metabolite 4-hydroxynonenal in human corneal epithelial cells. Graefes Arch Clin Exp Ophthalmol 258(8):1717–1725. 10.1007/s00417-020-04647-2 32445015 10.1007/s00417-020-04647-2

[CR41] Liu Y, Liang X, Dong W, Fang Y, Lv J, Zhang T, Fiskesund R, Xie J, Liu J, Yin X, Jin X, Chen D, Tang K, Ma J, Zhang H, Yu J, Yan J, Liang H, Mo S et al (2018) Tumor-repopulating cells induce *PD-1* expression in CD8+ T cells by transferring kynurenine and *AhR* activation. Cancer Cell 33(3):480–494.e7. 10.1016/j.ccell.2018.02.005 29533786 10.1016/j.ccell.2018.02.005

[CR42] Marsland BJ (2016) Regulating inflammation with microbial metabolites. Nat Med 22(6):581–583. 10.1038/nm.4117 27270775 10.1038/nm.4117

[CR43] Medzhitov R (2007) Recognition of microorganisms and activation of the immune response. Nature 449(7164):819–826. 10.1038/nature06246 17943118 10.1038/nature06246

[CR44] Nicholson JK, Holmes E, Kinross J, Burcelin R, Gibson G, Jia W, Pettersson S (2012) Host-gut microbiota metabolic interactions. Science 336(6086):1262–1267. 10.1126/science.1223813 22674330 10.1126/science.1223813

[CR45] Ori D, Kawai T (2023) Pathophysiological functions of self-derived DNA. Int Rev Immunol 42(4):274–286. 10.1080/08830185.2022.2070616 35499950 10.1080/08830185.2022.2070616

[CR46] Ouwerkerk PB, Hallard D, Verpoorte R, Memelink J (1999) Identification of UV-B light-responsive regions in the promoter of the tryptophan decarboxylase gene from *Catharanthus* roseus. Plant Mol Biol 41(4):491–503. 10.1023/a:1006321100550 10608659 10.1023/a:1006321100550

[CR47] Peterson LW, Artis D (2014) Intestinal epithelial cells: regulators of barrier function and immune homeostasis. Nat Rev Immunol 14(3):141–153. 10.1038/nri3608 24566914 10.1038/nri3608

[CR48] Piper CJM, Rosser EC, Oleinika K, Nistala K, Krausgruber T, Rendeiro AF, Banos A, Drozdov I, Villa M, Thomson S, Xanthou G, Bock C, Stockinger B, Mauri C (2019) Aryl Hydrocarbon Receptor contributes to the transcriptional program of *IL-10*-producing regulatory B cells. Cell Rep 29(7):1878–1892.e7. 10.1016/j.celrep.2019.10.018 31722204 10.1016/j.celrep.2019.10.018 PMC6856759

[CR49] Privitera G, Rana N, Scaldaferri F, Armuzzi A, Pizarro TT (2021) Novel insights into the interactions between the gut microbiome, inflammasomes, and gasdermins during colorectal cancer. Front Cell Infect Microbiol 11:806680. 10.3389/fcimb.2021.806680 35111698 10.3389/fcimb.2021.806680 PMC8801609

[CR50] Qin J, Li Y, Cai Z, Li S, Zhu J, Zhang F, Liang S, Zhang W, Guan Y, Shen D, Peng Y, Zhang D, Jie Z, Wu W, Qin Y, Xue W, Li J, Han L, Lu D et al (2012) A metagenome-wide association study of gut microbiota in type 2 diabetes. Nature 490(7418):55–60. 10.1038/nature11450 23023125 10.1038/nature11450

[CR51] Rank RG, Sanders MM, Patton DL (1995) Increased incidence of oviduct pathology in the guinea pig after repeat vaginal inoculation with the chlamydial agent of guinea pig inclusion conjunctivitis. Sex Transm Dis 22(1):48–54. 10.1097/00007435-199501000-00008 7709325 10.1097/00007435-199501000-00008

[CR52] Rohm TV, Meier DT, Olefsky JM, Donath MY (2022) Inflammation in obesity, diabetes, and related disorders. Immunity 55(1):31–55. 10.1016/j.immuni.2021.12.013 35021057 10.1016/j.immuni.2021.12.013 PMC8773457

[CR53] Saeedi BJ, Liu KH, Owens JA, Hunter-Chang S, Camacho MC, Eboka RU, Chandrasekharan B, Baker NF, Darby TM, Robinson BS, Jones RM, Jones DP, Neish AS (2020) Gut-resident Lactobacilli activate hepatic *Nrf2* and protect against oxidative liver injury. Cell Metab 31(5):956–968.e5. 10.1016/j.cmet.2020.03.006 32213347 10.1016/j.cmet.2020.03.006 PMC7329068

[CR54] Sen T, Cawthon CR, Ihde BT, Hajnal A, DiLorenzo PM, de La Serre CB, Czaja K (2017) Diet-driven microbiota dysbiosis is associated with vagal remodeling and obesity. Physiol Behav 173:305–317. 10.1016/j.physbeh.2017.02.027 28249783 10.1016/j.physbeh.2017.02.027 PMC5428886

[CR55] Sittipo P, Lobionda S, Lee YK, Maynard CL (2018) Intestinal microbiota and the immune system in metabolic diseases. J Microbiol 56(3):154–162. 10.1007/s12275-018-7548-y 29492872 10.1007/s12275-018-7548-y

[CR56] Smith, Macfarlane (1997) Formation of phenolic and indolic compounds by anaerobic bacteria in the human large intestine. Microb Ecol 33(3):180–188. 10.1007/s002489900020 9115181 10.1007/s002489900020

[CR57] de Vadder F, Kovatcheva-Datchary P, Goncalves D, Vinera J, Zitoun C, Duchampt A, Bäckhed F, Mithieux G (2014) Microbiota-generated metabolites promote metabolic benefits via gut-brain neural circuits. Cell 156(1–2):84–96. 10.1016/j.cell.2013.12.016 24412651 10.1016/j.cell.2013.12.016

[CR58] Wang L, Li C, Huang Q, Fu X (2020) Polysaccharide from Rosa roxburghii that fruit attenuates hyperglycemia and hyperlipidemia and regulates colon microbiota in diabetic db/db mice. J Agric Food Chem 68(1):147–159. 10.1021/acs.jafc.9b06247 31826616 10.1021/acs.jafc.9b06247

[CR59] Westbrook R, Chung T, Lovett J, Ward C, Joca H, Yang H, Khadeer M, Tian J, Xue Q-L, Le A, Ferrucci L, Moaddel R, Cabo R, Hoke A, Walston J, Abadir PM (2020) Kynurenines link chronic inflammation to functional decline and physical frailty. JCI Insight 5(16):e136091. 10.1172/jci.insight.136091 32814718 10.1172/jci.insight.136091 PMC7455140

[CR60] Whitehead TR, Price NP, Drake HL, Cotta MA (2008) Catabolic pathway for the production of skatole and indoleacetic acid by the acetogen *Clostridium drakei*, *Clostridium scatologenes*, and swine manure. Appl Environ Microbiol 74(6):1950–1953. 10.1128/AEM.02458-07 18223109 10.1128/AEM.02458-07 PMC2268313

[CR61] Wicherska-Pawłowska K, Wróbel T, Rybka J (2021) Toll-like receptors (TLRs), NOD-like receptors (NLRs), and RIG-I-like receptors (RLRs) in innate immunity. TLRs, NLRs, and RLRs ligands as immunotherapeutic agents for hematopoietic diseases. Int J Mol Sci 22(24). 10.3390/ijms222413397 10.3390/ijms222413397 PMC8704656 34948194

[CR62] Widner B, Werner ER, Schennach H, Wachter H, Fuchs D (1997) Simultaneous measurement of serum tryptophan and kynurenine by HPLC. Clin Chem 43:2424–2426 9439467

[CR63] Wright CJ, McKenna S, de Dios R, Boehmer BH, Nguyen L, Ghosh S, Sandoval J, Rozance PJ (2021) Lower threshold to NF-κB activity sensitizes murine β-cells to streptozotocin. J Endocrinol 249(3):163–175. 10.1530/JOE-21-0047 33764312 10.1530/JOE-21-0047 PMC8113150

[CR64] Wu Y, Dong L, Song Y, Wu Y, Zhang Y, Wang S (2022) Preventive effects of polysaccharides from *Physalis alkekengi* L. on dietary advanced glycation end product-induced insulin resistance in mice associated with the modulation of gut microbiota. Int J Biol Macromol 204:204–214. 10.1016/j.ijbiomac.2022.01.152 35108598 10.1016/j.ijbiomac.2022.01.152

[CR65] Xie Y, Wang H, Wu Y, Yin D, Wang Z, Huang Y (2011) Association of *APOE* polymorphisms and insulin resistance with TCM syndromes in type 2 diabetes patients with macroangiopathy. Mol Med Rep 4(6):1219–1223. 10.3892/mmr.2011.546 21822540 10.3892/mmr.2011.546

[CR66] Xu Y, Wang L, He J, Bi Y, Li M, Wang T, Wang L, Jiang Y, Dai M, Lu J, Xu M, Li Y, Hu N, Li J, Mi S, Chen C-S, Li G, Mu Y, Zhao J et al (2013) Prevalence and control of diabetes in Chinese adults. JAMA 310(9):948–959. 10.1001/jama.2013.168118 24002281 10.1001/jama.2013.168118

[CR67] Yan W, Hall AB, Jiang X (2022) *Bacteroidales* species in the human gut are a reservoir of antibiotic resistance genes regulated by invertible promoters. NPJ Biofilms Microbiomes 8(1):1. 10.1038/s41522-021-00260-1 35013297 10.1038/s41522-021-00260-1 PMC8748976

[CR68] Yang J-Y, Lee Y-S, Kim Y, Lee S-H, Ryu S, Fukuda S, Hase K, Yang C-S, Lim HS, Kim M-S, Kim H-M, Ahn S-H, Kwon B-E, Ko H-J, Kweon M-N (2017) Gut commensal *Bacteroides acidifaciens* prevents obesity and improves insulin sensitivity in mice. Mucosal Immunol 10(1):104–116. 10.1038/mi.2016.42 27118489 10.1038/mi.2016.42

[CR69] Yang W, Lu J, Weng J, Jia W, Ji L, Xiao J, Shan Z, Liu J, Tian H, Ji Q, Zhu D, Ge J, Lin L, Chen L, Guo X, Zhao Z, Li Q, Zhou Z, Shan G, He J (2010) Prevalence of diabetes among men and women in China. N Engl J Med 362(12):1090–1101. 10.1056/NEJMoa0908292 20335585 10.1056/NEJMoa0908292

[CR70] Yao M, Lu Y, Zhang T, Xie J, Han S, Zhang S, Fei Y, Ling Z, Wu J, Hu Y, Ji S, Chen H, Berglund B, Li L (2021) Improved functionality of *Ligilactobacillus salivarius* Li01 in alleviating colonic inflammation by layer-by-layer microencapsulation. NPJ Biofilms Microbiomes 7(1):58. 10.1038/s41522-021-00228-1 34244520 10.1038/s41522-021-00228-1 PMC8270932

[CR71] Yokoyama MT, Carlson JR (1979) Microbial metabolites of tryptophan in the intestinal tract with special reference to skatole. Am J Clin Nutr 32(1):173–178. 10.1093/ajcn/32.1.173 367144 10.1093/ajcn/32.1.173

[CR72] Youssef N, Sheik CS, Krumholz LR, Najar FZ, Roe BA, Elshahed MS (2009) Comparison of species richness estimates obtained using nearly complete fragments and simulated pyrosequencing-generated fragments in 16S rRNA gene-based environmental surveys. Appl Environ Microbiol 75(16):5227–5236. 10.1128/AEM.00592-09 19561178 10.1128/AEM.00592-09 PMC2725448

[CR73] Zelante T, Iannitti RG, Fallarino F, Gargaro M, de Luca A, Moretti S, Bartoli A, Romani L (2014) Tryptophan feeding of the *IDO1-AhR* axis in host-microbial symbiosis. Front Immunol 5:640. 10.3389/fimmu.2014.00640 25566253 10.3389/fimmu.2014.00640 PMC4266093

[CR74] Zhang G-D, Liu X-X, Liang J-L, Hu Q-M (2021) The distribution pattern of traditional Chinese medicine syndromes in 549 patients with type 2 diabetes. Diabetes Metab Syndr Obes 14:2209–2216. 10.2147/DMSO.S295351 34040406 10.2147/DMSO.S295351 PMC8139680

[CR75] Zhao C, Jia M, Song H, Yu Z, Wang W, Li Q, Zhang L, Zhao W, Cao X (2017) The E3 ubiquitin ligase *TRIM40* attenuates antiviral immune responses by targeting *MDA5* and *RIG-I*. Cell Rep 21(6):1613–1623. 10.1016/j.celrep.2017.10.020 29117565 10.1016/j.celrep.2017.10.020

[CR76] Zheng Z, Liu W, Ren Y, Li X, Zhao S, Yang H, Xiao Y (2021) *Allobaculum* involves in the modulation of intestinal *ANGPTLT4* expression in mice treated by high-fat diet. Front Nutr 8:690138. 10.3389/fnut.2021.690138 34095196 10.3389/fnut.2021.690138 PMC8171929

[CR77] Zhu L, Song Y, Liu H, Wu M, Gong H, Lan H, Zheng X (2021) Gut microbiota regulation and anti-inflammatory effect of β-carotene in dextran sulfate sodium-stimulated ulcerative colitis in rats. J Food Sci 86(5):2118–2130. 10.1111/1750-3841.15684 33884622 10.1111/1750-3841.15684

