# Supplementary material for: A study on the association between gut microbiota, inflammation, and type 2 diabetes
Source: Appl Microbiol Biotechnol. 2024 Feb 15;108:213. doi: 10.1007/s00253-024-13041-5 (PMC10869376; doi:10.1007/s00253-024-13041-5)
Supplement: Supplementary file 1 [file 253_2024_13041_MOESM1_ESM.pdf]

Supplementary Material

Article title: A study on the association between gut microbiota, inflammation and type 2 diabetes
Journal name: Applied Microbiology and Biotechnology
Author names: Nannan Liu, Xuehua Yan, Bohan Lv, Yanxiang Wu, Xuehong Hu, Chunyan Zheng, Siyu Tao, Ruxue Deng, Jinfang Dou, Binfang Zeng,Guangjian Jiang

Affiliation and e-mail address of the corresponding author: Binfang Zeng's affiliation is the Institute of Traditional Chinese Medicine, Xinjiang Medical University (Urumqi, China). His e-mail address is 2552721714@qq.com. Guangjian Jiang's affiliation is the Diabetes Research Center, Beijing University of Chinese Medicine (Beijing, China). Her e-mail address is bucmjiang@163.com.

Supplemental Table S1 lncRNA with a significant difference in the RLR signaling pathway in the peripheral blood of subjects between the YYLXD and N groups

**Transcript_id**	**Transcript_name**	**Gene_id**	**Gene_name**	**log2FoldChange**	***P*-value**
TCONS_00586946	*TCONS_00586946*	ENSG00000107201	*DDX58*	5.062084023	1.88E-05
TCONS 00586926	*TCONS 00586926*	ENSG00000107201	*DDX58*	4.417347163	5.38E-05
TCONS_00550234	*TCONS_00550234*	ENSG00000104365	*IKBKB*	3.998371075	0.009466996
TCONS_00586952	*TCONS_00586952*	ENSG00000107201	*DDX58*	3.865129342	0.028560856
TCONS 00586980	*TCONS 00586980*	ENSG00000107201	*DDX58*	3.818688468	0.021504751
ENST00000521661	*IKBKB-011*	ENSG00000104365	*IKBKB*	3.754843938	0.000679191
TCONS_00254134	*TCONS_00254134*	ENSG00000121060	*TRIM25*	3.677012506	0.014612619
TCONS_00474816	*TCONS 00474816*	ENSG00000145782	*ATG12*	3.495467799	0.000444055
TCONS_00254106	*TCONS_00254106*	ENSG00000121060	*TRIM25*	3.452468729	0.034978867
TCONS_00108436	*TCONS_00108436*	ENSG00000173039	*RELA*	3.394324349	0.036105905
TCONS_00487185	*TCONS_00487185*	ENSG00000156711	*MAPK13*	2.974051161	0.034441952
TCONS_00383108	*TCONS_00383108*	ENSG00000188130	*MAPK12*	2.916174415	0.040443485
TCONS_00098255	*TCONS_00098255*	ENSG00000160703	*NLRX1*	2.883854034	0.003582418
ENST00000456358	*TANK-012*	ENSG00000136560	*TANK*	2.853453	0.006678426
TCONS_00383132	*TCONS_00383132*	ENSG00000188130	*MAPK12*	2.709283944	0.000867903
ENST00000596788	*IRF3-026*	ENSG00000126456	*IRF3*	2.647490347	0.009296092
ENST00000507793	*ATG12-013*	ENSG00000145782	*ATG12*	2.633608794	0.007160155
TCONS_00212554	*TCONS_00212554*	ENSG00000083799	*CYLD*	2.532454728	0.040391424
TCONS_00400041	*TCONS_00400041*	ENSG00000168811	*IL12A*	2.440175869	0.027357981
TCONS_00254121	*TCONS_00254121*	ENSG00000121060	*TRIM25*	2.130250231	0.003075746
ENST00000556664	*NFKBIA-007*	ENSG00000100906	*NFKBIA*	1.674076867	pvalue
TCONS_00278778	*TCONS 00278778*	ENSG00000104825	*NFKBIB*	1.557847448	0.023304062
ENST00000531484	*RELA-005*	ENSG00000173039	*RELA*	1.499487879	0.038847181
ENST00000379594	*ATG12-007*	ENSG00000145782	*ATG12*	1.482421203	0.007282583
ENST00000557459	*NFKBIA-008*	ENSG00000100906	*NFKBIA*	1.043793209	0.002058171
ENST00000578982	*TBKBP1-003*	ENSG00000198933	*TBKBP1*	0.825048149	0.012876593
ENST00000455452	*OTUD5-002*	ENSG00000068308	*OTUD5*	0.688116963	0.021042119
TCONS_00238017	*TCONS_00238017*	ENSG00000198933	*TBKBP1*	0.62216985	0.013339504
ENST00000595240	*IRF3-016*	ENSG00000126456	*IRF3*	0.585434228	0.045298926
ENST00000392749	*TANK-002*	ENSG00000136560	*TANK*	0.58471599	0.019578641
ENST00000557389	*NFKBIA-004*	ENSG00000100906	*NFKBIA*	0.579358281	0.034410011
ENST00000555371	*NFKBIA-006*	ENSG00000100906	*NFKBIA*	0.562325296	0.037296522
TCONS_00177228	*TCONS_00177228*	ENSG00000100906	*NFKBIA*	0.495651601	0.037641099
ENST00000392745	*TRAF3-005*	ENSG00000131323	*TRAF3*	-0.705525355	0.027595725
ENST00000398568	*CYLD-005*	ENSG00000083799	*CYLD*	-0.888113777	0.036259594
TCONS_00254129	*TCONS_00254129*	ENSG00000121060	*TRIM25*	-1.007098216	0.049413708
ENST00000211287	*MAPK13-001*	ENSG00000156711	*MAPK13*	-1.239037477	0.022963615
ENST00000522133	*IKBKB-024*	ENSG00000104365	*IKBKB*	-1.314237311	0.026212885
TCONS_00339954	*TCONS_00339954*	ENSG00000115267	*IFIH1*	-1.800266486	0.042440272
TCONS_00480834	*TCONS_00480834*	ENSG00000050748	*MAPK9*	-2.814973816	0.009953183
ENST00000517917	*IKBKB-007*	ENSG00000104365	*IKBKB*	-4.398981095	0.023048407
ENST00000532096	*IRF7-011*	ENSG00000185507	*IRF7*	-4.869372573	0.037248306
TCONS 00261527	*RNF125-OT1*	ENSG00000101695	*RNF125*	-6.688274618	0.014594142
TCONS 00254096	*TCONS 00254096*	ENSG00000121060	*TRIM25*	-8 710138053	0 027114485

Supplemental Table S2 lncRNA with a significant difference in the RLR signaling pathway in the peripheral blood of subjects between the YYLXD and NYYLXD groups

Transcript_id	Transcript_name	Gene_id	Gene_name	log2FoldChange	*P*-value
ENST00000468831	*TANK-013*	ENSG00000136560	*TANK*	3.304594178	0.046550826
ENST00000508464	*ATG12-012*	ENSG00000145782	*ATG12*	2.697846994	0.029355416
ENST00000505252	*ATG12-005*	ENSG00000145782	*ATG12*	2.378547413	0.007077785
ENST00000509573	*TMEM173-014*	ENSG00000184584	*TMEM173*	-1.098006934	0.031280183
ENST00000596644	*IRF3-015*	ENSG00000126456	*IRF3*	-1.79440859	0.028704242
ENST00000522103	*IKBKB-016*	ENSG00000104365	*IKBKB*	-2.770298917	0.000123096
ENST00000517917	*IKBKB-007*	ENSG00000104365	*IKBKB*	-4.538106264	0.022955153
TCONS_00261527	*RNF125-OT1*	ENSG00000101695	*RNF125*	-5.940469618	0.008867485
